# Single-cell sequencing: a promising approach for uncovering the mechanisms of tumor metastasis

**DOI:** 10.1186/s13045-022-01280-w

**Published:** 2022-05-12

**Authors:** Yingying Han, Dan Wang, Lushan Peng, Tao Huang, Xiaoyun He, Junpu Wang, Chunlin Ou

**Affiliations:** 1 Department of Pathology, Xiangya Hospital, Central South University, Changsha, 410008 Hunan China; 2 Departments of Ultrasound Imaging, Xiangya Hospital, Central South University, Changsha, 410008 Hunan China; 3 Department of Pathology, School of Basic Medicine, Central South University, Changsha, 410031 Hunan China; 4 Key Laboratory of Hunan Province in Neurodegenerative Disorders, Xiangya Hospital, Central South University, Changsha, 410008 Hunan China; 5 National Clinical Research Center for Geriatric Disorders, Xiangya Hospital, Central South University, Changsha, China

**Keywords:** Single-cell sequencing, Tumor metastasis, Tumor heterogeneity, Tumor microenvironment, Artificial intelligence, Tumor marker, Targeted therapy

## Abstract

Single-cell sequencing (SCS) is an emerging high-throughput technology that can be used to study the genomics, transcriptomics, and epigenetics at a single cell level. SCS is widely used in the diagnosis and treatment of various diseases, including cancer. Over the years, SCS has gradually become an effective clinical tool for the exploration of tumor metastasis mechanisms and the development of treatment strategies. Currently, SCS can be used not only to analyze metastasis-related malignant biological characteristics, such as tumor heterogeneity, drug resistance, and microenvironment, but also to construct metastasis-related cell maps for predicting and monitoring the dynamics of metastasis. SCS is also used to identify therapeutic targets related to metastasis as it provides insights into the distribution of tumor cell subsets and gene expression differences between primary and metastatic tumors. Additionally, SCS techniques in combination with artificial intelligence (AI) are used in liquid biopsy to identify circulating tumor cells (CTCs), thereby providing a novel strategy for treating tumor metastasis. In this review, we summarize the potential applications of SCS in the field of tumor metastasis and discuss the prospects and limitations of SCS to provide a theoretical basis for finding therapeutic targets and mechanisms of metastasis.

## Background

During tumor metastasis, cancer cells from the primary tumors spread through the circulatory system or body cavities to colonize distant organs. These tumor cells then further colonize novel sites to form metastatic sites in distant organs [1, 2]. Various factors affect the occurrence and development of metastatic tumors, including genetic factors and in vivo microenvironment. The genotypes and phenotypes of metastatic tumor cells are often inconsistent with those of the cells present at the primary site. Tumor metastasis is an essential factor in cancer-related deaths and thus a major obstacle to tumor treatment. Significant progress has been made in the treatment of metastatic cancer treatment with the introduction of novel diagnostic techniques and treatment methods in recent years, yet the overall five-year survival rate in patients with metastatic cancer remains low [3]. This may be attributed to undetected tumor cell proliferation in early stages of cancer, as clinical symptoms often only appear in late stages [4–6]. Therefore, the identification of metastasis-related mechanisms, appropriate markers, and therapeutic targets is highly relevant in metastasis research.

With the development of high-throughput sequencing technologies and ChIP-seq platforms, single-cell sequencing (SCS) technology has enabled significant achievements in the diagnosis and treatment of various diseases, including metabolic, circulatory system, neurodevelopmental, and viral-infection-related diseases as well as cancer [7]. SCS techniques have been particularly instrumental for providing key insights in tumor metastasis research. For example, Perone et al. [8] compared genomes and transcriptomes of different cells using SCS and identified rare cell subsets, metastasis regulator key marker molecules, and their localization in metastatic tumors [9]. Bartoschek et al. [10] demonstrated the ability of SCS to predict and monitor tumor metastasis. Chen et al. [9] identified differentially expressed genes in tumor metastasis between primary and metastatic tumors using SCS and identified targets for metastatic cancer treatment. SCS techniques, in combination with artificial intelligence (AI), are used in liquid biopsy to identify circulating cells of tumors, thereby providing a theoretical basis for revealing novel metastasis-related targets. This review summarizes the recent applications of SCS in tumor metastasis research and discusses the prospects and limitations of SCS in this field. We expect this review to provide an important perspective for future metastasis research and the development of novel metastasis-targeting drugs.

## Single-cell sequencing (SCS)

### Development of SCS technology

**Fig. 1:**
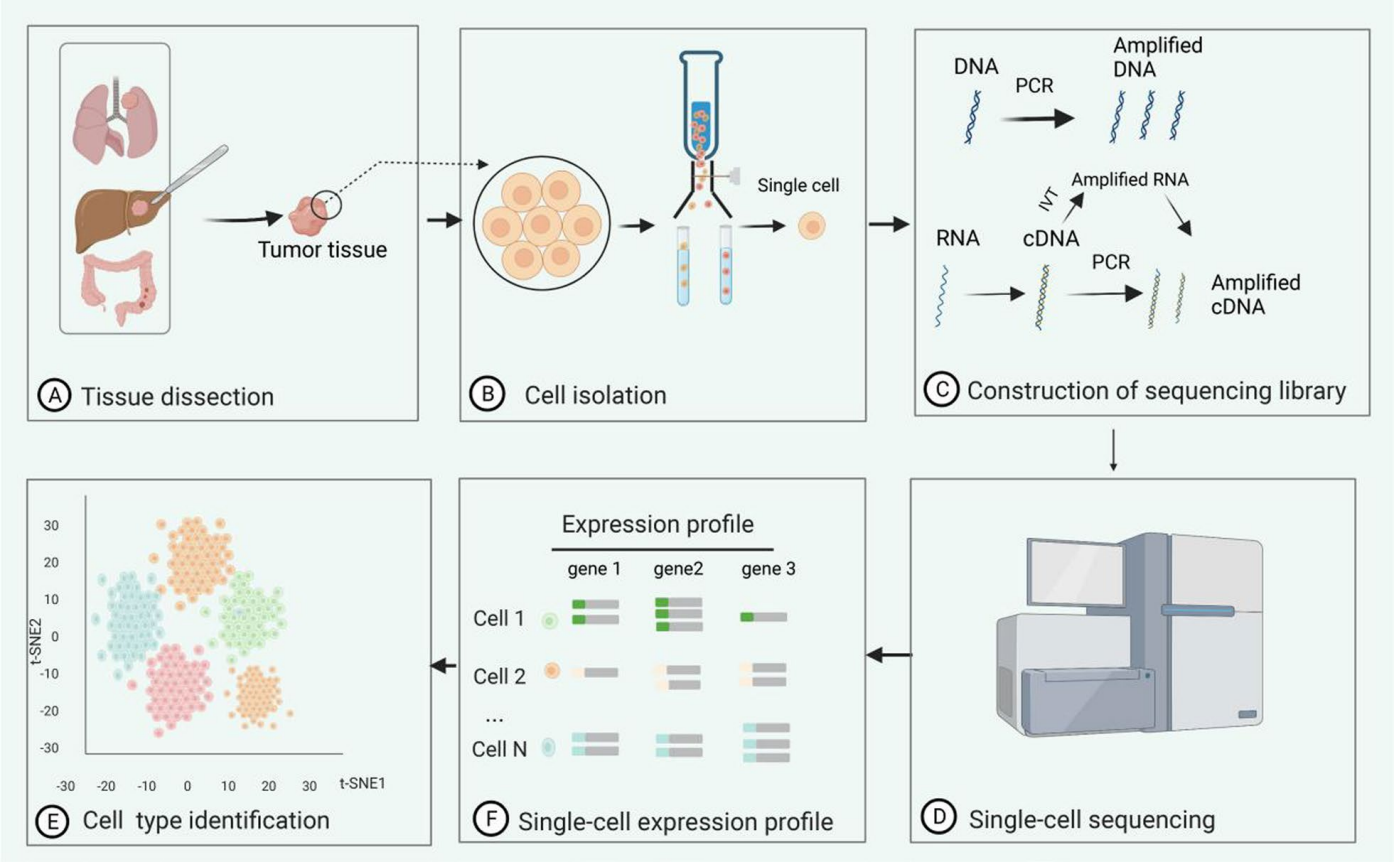
Schematic diagram of single-cell sequencing. First, solid tumor samples are processed to separate the surviving single cells **A**–**B**. Then, the single cells are lysed to obtain DNA or RNA, reverse-transcribed into cDNA, and then amplified to construct a sequencing library **C**. Once the sequencing library has been prepared, the critical single-cell sequencing steps are performed on a sequencing platform (**D**). After sequencing, it is necessary to visualize and interpret those data **E**–**F**

SCS is the study of the transcriptomics, genomics, and proteomics at the level of individual cells. In SCS, the whole genome and whole transcriptome of a single cell are amplified, and then high-throughput sequencing is performed, which reveals the structure and expression levels of genes in a single cell. Thus, even subtle differences between cells can be analyzed [7–12]. Typical SCS workflows include four main steps [13, 14] (Fig. 1). First, solid tumor samples are processed to separate surviving single cells (A-B). Then, the single cells are lysed to obtain the DNA or RNA, and then amplified to construct a sequencing library. However, RNA is first reverse-transcribed into cDNA(C). Once the sequencing library has been prepared, the critical SCS steps can be performed on a sequencing platform (D). After sequencing, it is necessary to visualize and interpret those data (E–F).

**Fig. 2:**
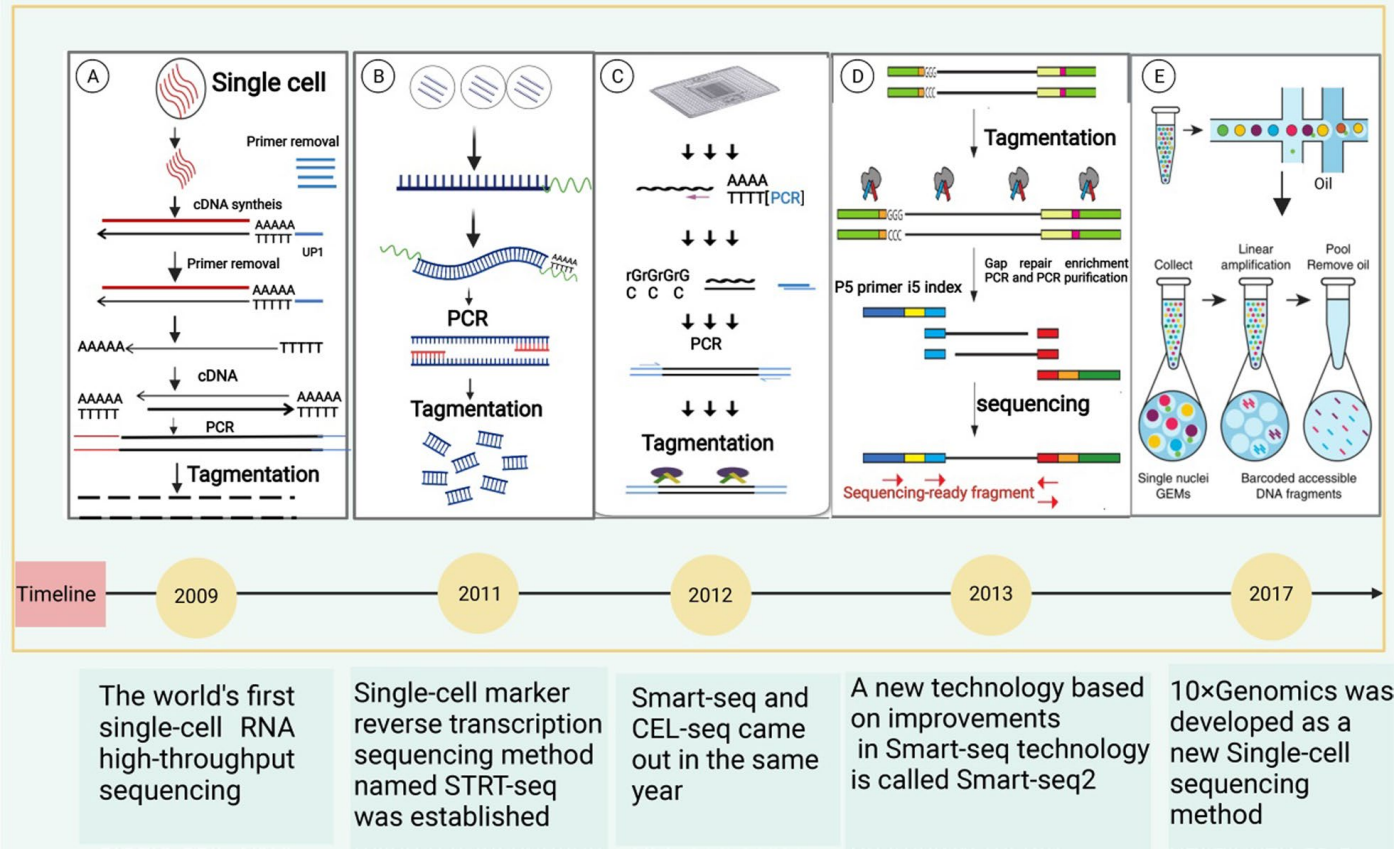
Timeline of milestones in single-cell sequencing technology. **A** Tang et al.[15] developed the first single-cell transcriptional sequencing technology, i.e., mRNA-seq in 2009. **B** Islam et al. [16] established a single-cell labeled reverse transcription sequencing method, i.e., STRT-seq in 2011. **C** Ramsköld et al.[17] developed a new single-cell sequencing technology Smart-seq in 2012; in the same year, Hashimshony et al. [25] developed single-cell RNA-Seq by multiplexed linear amplification named CEL-seq. **D** Picelli et al. [18] made some improvements to the Smart-seq technology in 2013, i.e., Smart-seq2. **E** 10 × Genomics technology was developed as a new single-cell transcriptome sequencing method in 2017

SCS has been widely used worldwide since the development of RNA sequencing (RNA-seq) technology in 2009 [15]. In 2011, Islam et al. [16] developed a single-cell labeled reverse transcription sequencing method named STRT-seq. In 2012, a new SCS variant called Smart-seq was developed [17], which was improved in 2013 by Picelli et al. [18] who also developed Smart-seq2. In 2017, 10 × Genomics was developed as a novel single-cell immune repertoire sequencing method. This technique introduced significant advancements in sequencing efficiency in the context of technical- and application-oriented aspects of SCS. Azizi et al. [19] analyzed 45,000 immune cells from eight primary breast cancer patients using 10 × Genomics SCS platforms and revealed detailed phenotypes of immunocytes in the tumor microenvironment (TME) (Fig. 2).

### Classification of SCS technology

Based on the analysis of the obtained sequencing data, SCS can be divided into three main types, i.e., single-cell DNA sequencing (scDNA-seq), single-cell RNA sequencing (scRNA-seq), and single-cell immune repertoire sequencing (scIR-seq). The scDNA-seq and scRNA-seq are two commonly used single-cell sequencing technologies, of which two have subtle differences in their operating procedures. scDNA-seq amplifies the whole-genome DNA of isolated single cells, while scRNA-seq first reverse-transcribes the whole-transcriptome RNA of a single cell into cDNA, which is then amplified; after analysis, the visualized data reveal the cell population differences and cell evolutionary relationships [7, 9–12, 20–22]. scIR-seq has gained significant attention in recent years due to the development of tumor immunotherapy technology. In scIR-seq, T/B lymphocytes are the research target, and multiplex PCR technology/5'RACE is used to amplify the complementarity determining regions (CDR3 regions) that determine the diversity of B cell receptors (BCRs) or T cell receptors (TCRs). This is then combined with high-throughput sequencing technology to comprehensively evaluate the diversity of the immune system and examine the relationship between the immune repertoire and disease [23, 24].

To date, dozens of single-cell transcriptome sequencing methods have been developed, each with its own characteristics and advantages/disadvantages. Commonly used SCS analysis methods are mainly divided into seven types, i.e., two low-flux plate-based methods (Smart-seq2 [18] and CEL-Seq2 [25]) and five high-throughput methods (10 × Chromium [26], Drop-seq [27], Seq-Well [28], InDrops [29], and Sci-RNA-seq [30]). No single sequencing platform is suitable for all research objectives [18–35] (Table 1). Researchers should choose a suitable sequencing platform in view of their respective research purposes.

**Table1:** Advantages and disadvantages of the common SCS methods

Method	Flux	Advantages	Disadvantages	Ref
Smart-seq2	Low	a. High sensitivity, high transcription coverage b. Cell capture visualization c. Analysis of rare cell populations	a. No early multiplexing b. Longer cycle	[18, 28]
CEL-Seq2	Low	a. Higher sensitivity, lower cost b. Lower hands-on input	a. Strong 3' preference b. High-abundance transcripts are preferentially amplified	[25, 34 30]
10x- Chromium	High	a. Less time-consuming and low technical noise b. Analysis of rare cell population	a. There are too many steps for DNA library construction b. Higher sample requirement	[18, 19, 24, 26]
Drop -seq	High	a. Low cost and fast b More effective	Lower cell capture efficiency	[27, 29 35]
Seq-Well	High	Easy-to-use, portable, low cost b. Efficient cell lysis and transcriptome capture	a. Lower cell capture efficiency	[28, 33 35]
In Drops	High	a. Lower cost b. Strong cell capture and simplification capabilities	a. Extremely lower cell capture efficiency	[29, 35]
Sci-RNA-seq	High	a. Minimize perturbation to RNA integrity	a. Some cell types cannot be defined	[30]

### Application of SCS in human diseases

SCS is widely used to study human diseases, including metabolic, circulatory, neurodevelopmental, and viral-infection-related diseases as well as cancer [36–45] (Table 2). For example, Farrell et al. [39] comprehensively analyzed gene expression profiles in individual brain cells of Alzheimer’s patients using SCS and identified potentially disease-related signaling pathways, thereby providing a theoretical basis for drug development. Furthermore, Wilk et al. [43] used SCS to analyze peripheral blood mononuclear cells (PBMCs) of seven critically ill patients hospitalized for the treatment of coronavirus disease 2019 (COVID-19) and constructed a cell map of the peripheral immune response in these patients in an effort to better understand the immune cell composition in COVID-19 patients and to assist the development of a COVID-19 vaccine. In human cancers, Kim et al. [44] characterized 208,506 cells based on the single-cell transcriptome profile of metastatic lung adenocarcinoma and identified a cancer cell subtype deviating from the normal differentiation trajectory and dominating the metastatic stage. Thus, the application of single-cell sequencing technology is quite extensive and can benefit human beings in various aspects.

**Table 2:** Application of SCS in human diseases

Disease type	Sample type	Number	Detection Method	Conclusion	Ref
Diabetic kidney	Cells	–	scRNA-seq	Revealed the dynamic changes of gene expression in the diabetic kidney	[36]
Rheumatoid arthritis	Cells	51 patients	scRNA-seq	Discovered the key mediators of the pathogenesis of RA	[37]
Heart injury	Cells	30,000 cells	scRNA-seq	Provided an in-depth analysis of the entry points of cardiac homeostasis, inflammation, fibrosis, repair and regeneration	[38]
Alzheimer's disease	Cells	38,731 cells	scRNA-seq	Discovered potential signaling pathways in Alzheimer's disease	[39]
Biliary atresia	Cells	–	scRNA-seq	Demonstrated that B cell modification therapy can alleviate liver pathology	[40]
Cirrhosis	Cells	4076 cells	10 × scRNA-seq	Displayed the development of novel therapeutic strategies to target the most dysfunctional liver ECs	[41]
Placenta	Cells	—	scRNA-seq	Revealed the unprecedented depth for the investigation of cell type-specific gene expression patterns in the placenta	[42]
SARS-CoV-2	Cells	7 patients	scRNA-seq	Provided potential therapeutic targets for cell resistance	[43]
Lung adenocarcinoma	Cells	208,506 cells	scRNA-seq	Identified a cancer cell subtype deviating from the normal differentiation trajectory	[44]
Pan-cancer	Cells	21 types of cancer cells	scRNA-seq	Revealed distinct patterns of T cell composition	[45]

## Tumor metastasis

**Fig. 3:**
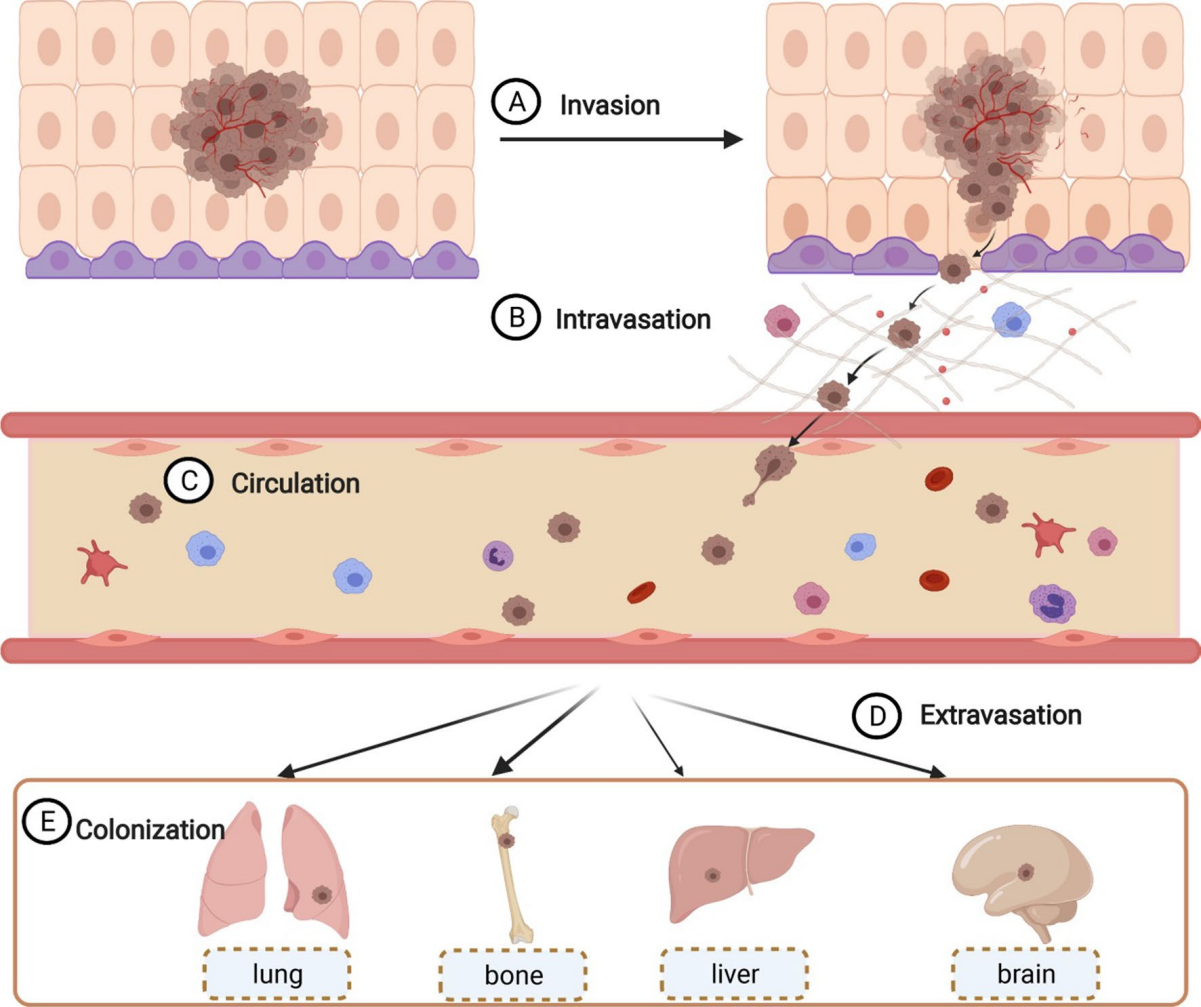
The process of tumor metastasis. Tumor metastasis is complex and involves multiple steps, i.e., local invasion (**A**), intravasation (**B**), circulation (**C**), extravasation (**D**), and seeding (**E**)

Malignant tumor cells disseminate from the primary tumor site through the lymphatics, blood vessels, or body cavities to other body parts during metastasis [1, 2]. This is a complex and multi-step process (Fig. 3) which often includes local invasion, intravasation, circulation, extravasation, and seeding [46–48].

Infiltration into adjacent tissues and metastasis to distant organs are major features of malignant tumors. Tumor metastasis also involves the activation and inactivation of protooncogenes and suppressor oncogenes, respectively, which regulate different signal transduction pathways. For instance, MAPK[49], JAK-STAT [50, 51], Wnt [52–55], and other signaling pathways are closely related to tumor metastasis. The components of tumor cells and their surrounding environment also change before or during tumor metastasis. Primary and metastatic tumors have been shown to be indeed significantly different from each other in terms of tumor heterogeneity, drug resistance, and TME. A study on prostate cancer revealed similar genetic profiles of primary and metastatic sites, yet additional mutations in the metastatic site were detected as well, indicating specific intratumoral heterogeneity [56]. Moreover, the activity of P-glycoprotein (P-Gp), a multidrug resistance (MDR) efflux transporter, is increased during epithelial-mesenchymal transition (EMT) in cancer progression [57, 58]. When P-Gp expression is significantly reduced, cell migration and invasive abilities of MDR cells decrease significantly [59]. In fact, the TME in the metastatic site is selectively activated prior to metastasis to create favorable tumor growth conditions [60, 61]. The microenvironment around the primary tumor also changes simultaneously prior to metastasis, initiates the transition of cells to obtain certain unique biological characteristics, and thus promotes metastasis [62]. Components and intercellular communication in TME can also promote tumor formation, metastasis, and drug resistance [63–66], such as tumor-related macrophages (TAMs) [67, 68], cancer-related fibroblasts (CAFs)[69–71], and EMT [72, 73]. Overall, tumor metastasis is closely related to tumor heterogeneity, tumor drug resistance, and TME.

## SCS and tumor metastasis

**Fig. 4:**
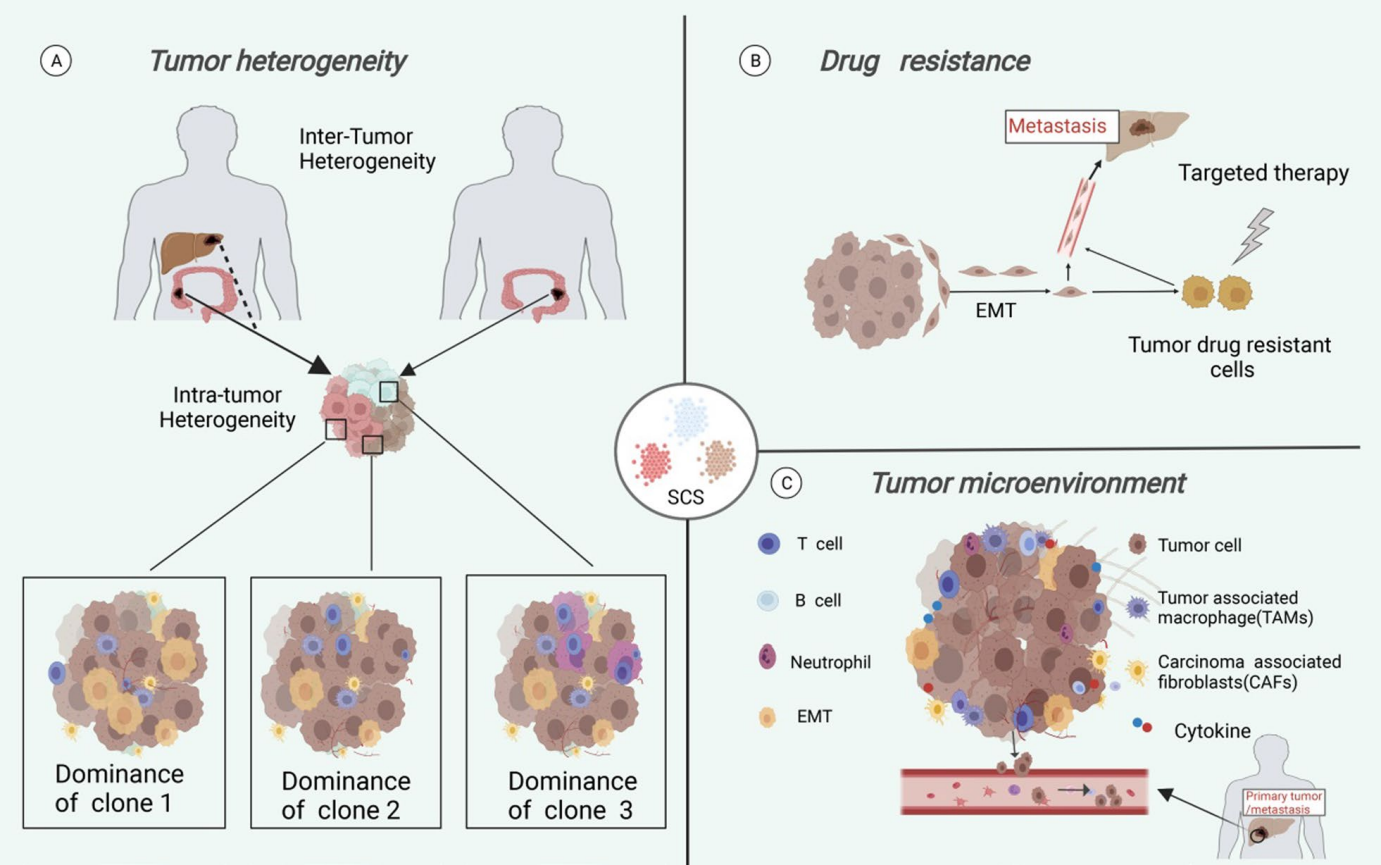
SCS and tumor metastasis. SCS technology can be used to examine the relationship between tumor metastasis and tumor heterogeneity (**A**), tumor drug resistance (**B**), and tumor microenvironment (**C**)

SCS technology can be used to examine the relationship between tumor metastasis and tumor heterogeneity, tumor drug resistance, and TME (Fig. 4), providing a good platform for revealing the mechanism of tumor metastasis and proposing new strategies for treating tumor metastasis.

### SCS and tumor metastasis-associated heterogeneity

Heterogeneity between and within tumors often occurs during tumor development. Thus, genotype and phenotype variation exists in the same tumor or in different tumors. More directly, there is a difference between primary and metastatic sites [74]. Tumor heterogeneity is an important characteristic of malignant tumors that renders basic research, clinical diagnosis, and treatment of tumor metastasis difficult. During metastasis, tumor heterogeneity reflects differences in genetic, epigenetic, metabolic, and immune responses between primary sites and metastases, such as immune infiltration degree and immune and tumor cell types [75–77]. Furthermore, tumor cells in the metastatic site often exhibit specific driver mutations after metastasis. For example, a mutation in driver genes has been observed in advanced recurrent metastatic breast cancer, which has not been found in early primary breast cancer [8]. In addition to the spatial heterogeneity mentioned above, heterogeneity also exists in the time dimension in metastasis. In other words, there are also differences in heterogeneity of the same tumor at different time points during metastasis. SCS can be used to reveal genetic, transcriptional, and metabolic characteristics of tumor metastasis-related heterogeneity at the single-cell level.

The expression levels of genes involved in different metastasis-related biological processes vary continuously during metastasis. Toward this end, SCS is useful to understand the mechanisms underlying gene expression dynamics, and reveal genetic, transcriptional, and metabolic heterogeneity between primary and metastatic tumors [78]. Okamoto et al. [79] analyzed organoids originating from patients with primary and metastatic colorectal cancer using the SCS technology and found that expression levels of differentiated cell marker genes were inconsistent between primary and metastatic lesions, indicating differences in the genetic composition of metastatic lesions. Davis et al. [80] also used SCS to study patient-derived xenograft models of breast cancer and found that both primary tumor and micro-metastasis cells display transcriptional heterogeneity, yet micro-metastasis cells harbor a distinct transcriptional profile. There are also significant differences in the metabolic expression profiles of primary breast cancer and pulmonary metastases. Pharmacological inhibition of oxidative phosphorylation dramatically attenuated metastatic seeding in lungs, demonstrating the functional importance of oxidative phosphorylation in metastasis. Another study also revealed metabolic heterogeneity between primary and metastatic tumors in pancreatic cancer [81]. As the most malignant osteogenic tumor, osteosarcoma easily metastasizes to the lungs. SCS analysis of primary and pulmonary metastatic osteosarcoma revealed lower osteoblast infiltration and inflammatory FABP4^+^ macrophages in pulmonary metastatic osteosarcoma. The difference in the types and proportions of immune cells between the two osteosarcoma types indicates tumor heterogeneity [82]. Using SCS, Ni et al. [83] found that different CTCs from the same patient showed a highly consistent copy number change pattern throughout the whole genome, which was highly consistent with the copy number change patterns of the metastatic tumor tissue of the same patient. This phenomenon was observed in patients with small-cell lung cancer and lung adenocarcinoma. This highly consistent copy number change pattern observed in CTC for the first time is likely to change the traditional understanding of tumor consistency.

Tumors also show heterogeneity in both time and space. SCS can accurately detect dynamic changes in heterogeneity among tumor cells across time and different spatial positions [84]. Indeed, multi-region SCS analyses of lung cancer showed a considerable degree of intra-tumor heterogeneity of immune-related genes in spatial [85] and temporal [86] dimensions. A similar analysis of different metastases in ovarian cancer patients showed that immune and interstitial components of the different metastatic sites have considerable spatial heterogeneity [87, 88]. An SCS analysis of cancer stem cells (CSCs) from a pair of primary and metastatic sites of collecting duct renal cell carcinoma (CDRCC) showed that CSCs can transform into primary and metastatic CDRCC cells in a spatiotemporal manner [89]. SCS is also used to reconstruct the history of tumors and tumor subclone development, identify cell subtypes that are likely to metastasize, and possibly discover genes that drive metastasis and subclone development. Puram et al. [90] performed an SCS analysis on primary and lymph node metastatic cells from 18 patients with head and neck squamous cell carcinoma. Based on different types of immune and stromal cells in the TME, molecular subtypes in the head and neck squamous cell carcinoma were redefined. Specifically, the authors identified a group of frontal tumor cells with EMT characteristics closely related to lymph node metastasis. Triple-negative breast cancer (TNBC) is a unique subtype of breast cancer that is negative for estrogen receptor (ER), progesterone receptor (PR), and human epidermal growth factor (HER2), has a wide range of intra-tumor heterogeneity, and has a poor prognosis. Karaayvaz et al. [91] performed cluster analysis of gene expression profiles obtained from SCS analysis on six main cells with TNBC > 1500 and showed the existence of different tumor cell subtypes, including those with metastasis and treatment resistance. Subtypes that easily metastasize can be identified using SCS. Yates et al. [92] sequenced whole genomes of samples from metastatic breast cancer patients and showed that most of the distant metastases obtained unseen driver gene mutations compared to the primary tumors. SCS can also be used to construct a tumor transfusion spectrum and determine metastasis onset time. Navin et al. [93] used SCS to analyze 100 single cells of primary breast cancer and its liver metastasis. According to their results, a single clonal amplification formed the primary tumor, initiated metastasis, and formed unexpectedly genetically “fake diploid” cell subgroups that did not reach the transfer site. The primary site was also found to be similar to the transfer site in terms of the SCS copy number variant data. This finding supports the hypothesis that transfer occurs during the late stages of clonal evolution.

In summary, the findings discussed above show that SCS can reveal intra- and inter-tumor heterogeneity at the single-cell level, identify characteristics and states of cells, and identify potential key factors for tumor occurrence and metastasis to guide the development of precise treatment options, draw an overall map of the tumor, and track the lineage of metastasized cells.

### SCS and tumor metastasis-associated drug resistance

Chemotherapy is currently a major treatment option for malignant tumors. However, chemotherapy drugs may alter the phenotype of tumor cells and lead to drug resistance. Many recent studies have shown a close association between metastasis and drug resistance. For example, CSCs can induce drug resistance and metastasis. CSCs are first transformed into primary and then metastatic cells. During metastasis, CSCs pass through EMT and mesenchymal–epithelial transition (MET) and form a distant metastatic site [89]. Heterogeneity associated with tumor metastasis is the main driving force for tumor resistance [94]. The tumor genes of cells in metastatic lesions often include mutations, which result in drug resistance following metastasis. Jordan et al. [95] detected rare CTCs from the blood of primary breast cancer patients and identified dynamic gene expression profiles in metastatic breast cancer that promote disease development and resistance to treatment. For instance, the activity of P-Gp increases during metastasis-related EMT [57, 58], whereas significant decreases in P-Gp expression result in corresponding decreases in migration and invasion abilities of MDR cells [59]. Thus, tumor metastasis mechanisms can be further elucidated by analyzing the formation of drug resistance in cancer cells.

Interaction between tumor drug resistance and metastasis has been shown in recent years. Long-term exposure to chemotherapeutic drugs greatly promotes tumor invasion and metastasis [96, 97], and drug-resistant cells are more likely to metastasize [98–100]. SCS can be used to study and analyze drug-resistant tumor cells to avoid the interference of tumor heterogeneity, providing a new perspective for exploring tumor metastasis. Prostate cancer patients respond to androgen receptor (AR) inhibitors to a certain extent. Miyamoto et al. [101] performed an SCS analysis of single CTCs in 17 prostate cancer patients and found that atypical Wnt signals were enriched in CTCs of drug-resistant rather than untreated patients. Expression of Wnt signaling components in prostate cancer was shown to promote the metastasis of prostate cancer [102]. Accordingly, drug-resistant cells were found to be more likely metastasized. Expression levels of several EMT-related genes were also previously shown to be altered in drug-resistant human breast cancer (MCF-7) cells [103]. A large-scale SCS analysis further showed that chemotherapy increased the metastatic ability of breast cancer cells [100]. Lee et al. [104] performed an SCS analysis of untreated paclitaxel-resistant metastatic breast cancer and found specific transcriptional profiles in the cell population in addition to a specific RNA variant in drug-resistant cells. This variant is involved in microtubule stabilization and cell adhesion, which indirectly indicates that drug-resistant cells are more likely to develop tumor metastasis. In another breast cancer study, various single-cell gene expression profiles in chemotherapy-treated cell lines showed that EMT-related genes were upregulated in drug-resistant cells, mainly by the *LEF1* gene. EMT is generally considered a key factor for tumor metastasis and drug resistance. Thus, chemotherapy increases tumor metastasis risk, and enhanced metastatic ability of drug-resistant tumor cells is associated with the upregulation of EMT-related genes [105]. Nath et al. [106] also reported high drug resistance due to EMT-related genes in addition to high expression of the double multidrug resistance (*MDR1*) gene. Tumor drug resistance is mediated by cell proliferation, apoptosis, invasion, and migration [107]. Single-cell transcriptional map analysis of tumor tissue samples from six patients with TNBC showed subclonal heterogeneity among malignant tumor cells shared by different patients, which is characterized by drug resistance and metastasis [91]. Briefly, upregulation of EMT-related proteins is often accompanied by an increase in tumor drug resistance and high rate of proliferation and metastasis of drug-resistant cells. Thus, enrichment of drug-resistant tumor cells can lead to tumor metastasis. Hjortland et al. [98] conducted a genome-wide single-cell analysis of chemotherapy-resistant metastatic cells of gastroesophageal adenocarcinoma, analyzed the molecular characteristics of drug-resistant metastatic cells, and identified markers responsible for malignant progression and potential therapeutic targets. Another study discussed the feasibility of SCS in monitoring the emergence of drug-resistant cell clones in tumors [108]. CSCs are capable of undergoing cell division and, therefore, give rise to heterogeneity in the tumor, playing a crucial role in tumorigenesis. Characterization of CSCs properties can provide important information regarding tumor metastasis and drug resistance [109, 110]. Chen et al. [9] enriched metastatic breast cancer cells with microfluidics, and identified differentially expressed genes in metastatic cells via SCS. Migrating cells were found to have the overall characteristics of EMT and CSCs, yet different properties with respect to mitochondrial morphology, oxidative stress, and proteasome regulators, revealing potential vulnerability and unexpected consequences of drug treatments. Franken et al. [111] performed an SCS analysis of 46 metastatic breast cancer patients, focusing on the *ESR1* gene of CTC. The results showed that *ESR1* mutations were only detected in metastatic foci, but not in primary tumor tissue samples. Moreover, *ESR1* mutations only appeared in patients who received estrogen deprivation therapy. The authors thus concluded that the newly discovered mutation might lead to targeted drug resistance and tumor metastasis.

These studies demonstrate that SCS can be used to analyze drug-resistant cell subtypes in tumors, detect key drug-resistant genes, discover potential drug targets, and provide a theoretical basis for improved targeted therapy for drug-resistant tumors.

### SCS and tumor metastasis-associated tumor microenvironment

The relationship between TME and tumors is often described with the terms “seed” (the tumor cell) and “soil” (TME). TME indeed plays an indispensable role in tumor metastasis [112, 113]. The driving factors of mutant genes with proliferation and invasion in tumor cells are in line with the importance of TME in this regard [56]. TME-related cells and molecules also play an important role in tumor metastasis, such as fibroblasts, tumor-associated macrophages, immune cells, and cytokines. The “premetastatic niche” concept first proposed by Psaila et al. [114] denotes that the primary tumor will release a series of signal molecules that change the local microenvironment around the metastasis site prior to the arrival of the tumor cells. Thus, key regulatory molecules that play roles in tumor occurrence, development, and metastasis can be identified by clarifying the relationship between tumor metastasis and TME and analyzing changes in TME before and after tumor metastasis.

SCS can also be used to investigate the heterogeneity between primary tumors and metastatic TMEs based on analysis of the microenvironment composition and accompanying molecular changes. Arvanitis et al. [115] studied the characteristic structures of the blood–brain barrier (BBB) and blood–tumor barrier (BTB) of primary brain tumors and brain metastases by SCS and found structural and functional heterogeneity between the metastatic and primary tumors in the microenvironment. This in-depth study of BBB/BTB structure and tumor cell subtypes between primary and metastatic tumors thus enabled a better understanding of tumor progression and metastasis as well as identification of targeted immunotherapy strategies. Lee et al. [116] analyzed the TME of metastatic colorectal cancer by using the SCS technology and revealed the diversity of cell components of CRC molecular subtypes, their dynamic relationship, and the TME landscape of CRC. Robinson et al. [117] performed an SCS-based analysis of the whole exons and transcriptome of 500 metastatic and non-cancerous tissue samples from metastatic cancer patients. These studies provided detailed analyses of genomes and immune responses in metastatic cancer tissues, indicating the complex molecular landscape of metastatic tumors. SCS can also be used to study the metastasis-related genes or cell types in the TME, including (but not limited to) CAFs and TAMs. Li et al. [118] analyzed the transcriptional heterogeneity of colorectal tumors and their microenvironment via SCS and found two different CAF subtypes. The expression of EMT-related genes increased only in the CAF subgroup of tumor tissues, indicating a possible role of CAF in tumor metastasis and invasion ability. Bao et al. [119] analyzed TNBC using SCS and characterized the heterogeneity between and within tumors. The authors found that M2-like TAMs accounted for the majority of macrophages in tumor-infiltrating immune cells and showed immunosuppressive characteristics. In contrast, M2-like TAMs have been previously shown to be closely related to tumor metastasis [67–69]. Winterhoff et al. [120] performed enzymatic digestion of serous ovarian cancer tissue to remove immune cells, identified two subsets of epithelial and tumor-related stromal cells among 66 cells via STS, and described the characteristics of these two subsets. Accordingly, the expression of EMT-related genes was found to increase in stromal cell subsets, which provided a new perspective on invasion and metastasis of serous ovarian cancer.

The importance of the degree of immune infiltration and type of immune cells in the TME in regulating tumor progression has also been emphasized recently. Metastatic sites often show different immune cell enrichment patterns [121]. Understanding the composition and function of the primary tumor immune microenvironment (TIME) and its metastasis is thus a prerequisite for successful cancer immunotherapy. SCS enables identification of the heterogeneity of TIME and specific characteristics of immune cells, especially those of T cells, to design better immunotherapy strategies. Zhang et al. [122] analyzed liver metastasis samples from CRC patients and adjacent tissues by SCS and revealed heterogeneity of TIME in liver metastasis from colorectal cancer. Identification of immune cell subtypes in this study allowed analysis of tumor-infiltrating T cell subsets and, ultimately, highlighted the role of granulocytes in TIME. The technology also allows identification of unique metastasis-related immune cell subsets and potential immunotherapy targets.

These studies show that SCS can be used to map the microenvironment of metastatic cancer cells and analyze obvious structural and functional heterogeneity of TME between metastatic and primary tumors in order to identify metastasis-related genes or cell types. SCS is also particularly helpful to explore potential new targets for tumor immunotherapy via identification of the relationship between tumor metastasis and immune cells.

## Application of SCS in cancer treatment

**Fig. 5:**
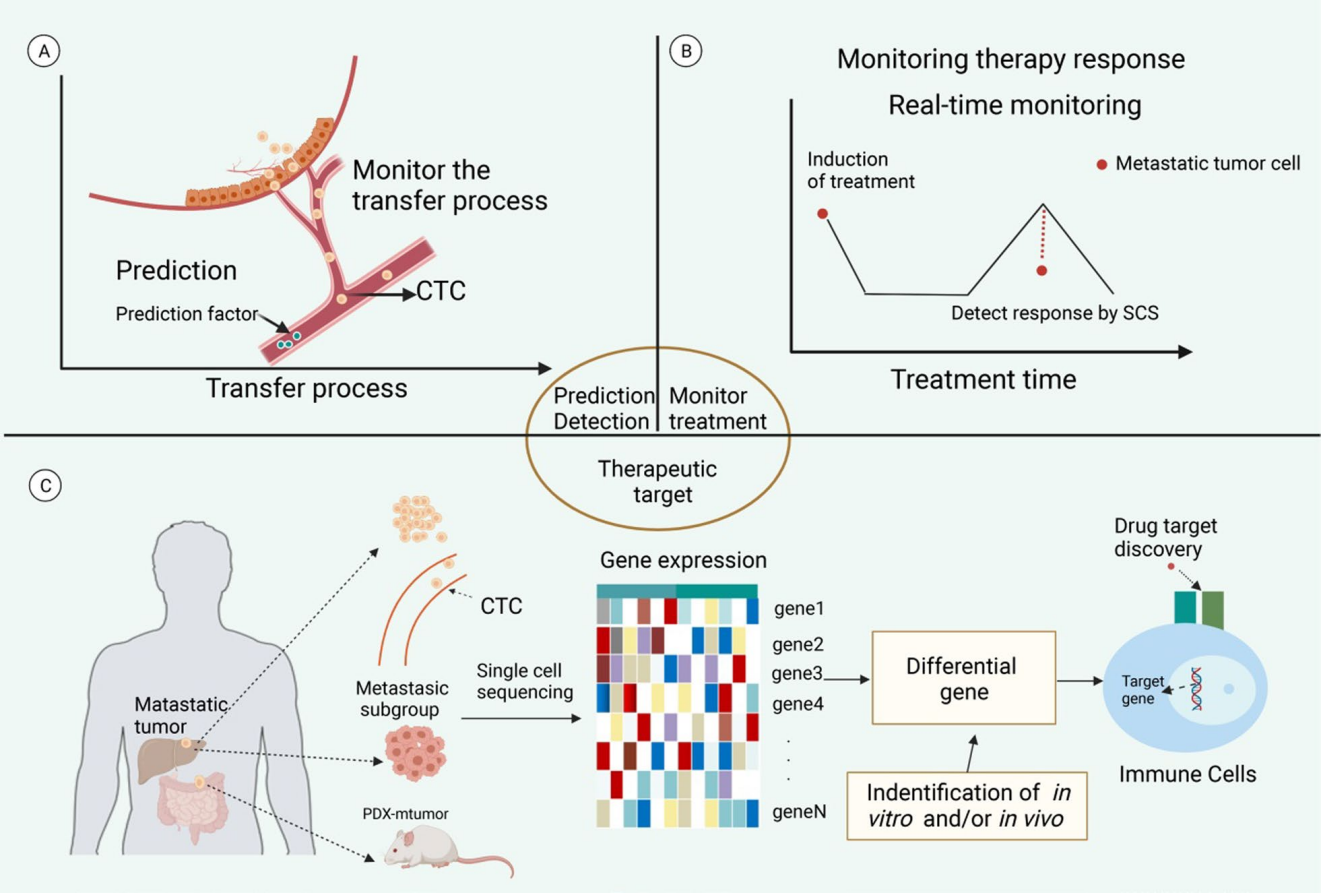
Application of single-cell sequencing (SCS) for treating tumor metastasis. **A** SCS can be used for predicting and monitoring tumor metastasis. **B** SCS can be used for clarifying the mechanism of tumor metastasis and provide treatment targets. **C** SCS can be used for monitoring and predicting the treatment response and optimizing the treatment strategies

SCS has excellent application potential in the treatment of metastatic cancer and can be used for purposes such as prediction and monitoring of tumor metastasis, clarification of metastasis mechanisms, identification of therapeutic targets, monitoring and prediction of the therapeutic response, and optimization of treatment strategies (Fig. 5).

### Monitoring of tumor metastasis

Current knowledge of tumor metastasis mechanisms is highly limited, and many questions lack clear answers, including which cell subtypes or clones in primary tumors can spread and metastasize, how many times tumor cells can metastasize to distant organs, and whether the whole process of tumor metastasis can be monitored. With recent developments in SCS, progress has been achieved in finding answers to these open questions.

SCS can predict whether tumors will metastasize and can help identify markers for predicting metastasis. For example, an SCS study of CAFs in 190 cases of distant metastatic breast cancer tissues by Bartoschek et al. [10] showed that the expression of marker genes in different CAF subtypes indicates whether human breast cancer will metastasize, confirming the association of CAF subtypes with metastasis and spread of tumors. Consistent with the Bartoschek et al.[10] study, in an SCS study of pancreatic ductal carcinoma, the primary tumor was shown to have a higher metastatic capacity if the TME is rich in new CAF subtypes [123]. Puram et al. [90] analyzed primary and metastases of the head and neck squamous cell carcinoma by SCS and showed some distinct characteristics related to partial EMT (p-EMT). Specifically, the level of p-EMT in cells located at the front edge of the primary tumors can be used as a predictive marker of tumor metastasis. SCS of tumors at different time points can also be used to monitor rare mutations during tumor development and progression, such as gaining the ability to invade and metastasize. To this end, Davis et al. [80] developed a novel method to monitor global transcriptome changes of several metastatic cells in the process of breast cancer metastasis based on SCS and a patient-derived breast cancer xenotransplantation (PDX) model to identify whether the tumor is in the process of metastasis by monitoring the changes in the cell transcriptome and predicting adverse survival of the patients. Interestingly, such a systematic genome analysis of metastatic prostate cancer pedigree was found to help identify whether metastasis spreads only once or whether multiple metastases will be found [124].

### Searching for potential therapeutic targets

SCS can be used to analyze heterogeneity of tumor cells during metastasis by assisting the discovery of genes and cell subsets related to metastasis and identification of potential therapeutic targets [9, 41, 80, 122, 124–132] (Table 3). For instance, Kim et al. [41] used SCS to analyze single-cell transcriptome profiles of metastatic lung adenocarcinoma cells. In addition to identifying a cancer cell subtype deviating from the normal differentiation trajectory, they also identified genes related to the progression and metastasis of lung adenocarcinoma and highlighted that ts2-specific related genes were related to tumor progression and metastasis. This finding indicated that ts2-specific related genes could represent new therapeutic targets for metastatic lung adenocarcinoma. Chen et al. [9] enriched metastatic breast cancer cells with microfluidics, and then identified differentially expressed genes in metastatic breast cancer cells using SCS. The results revealed genes that migrated with breast cancer cells as potential prognostic biomarkers and therapeutic targets for treatment of metastatic breast cancer. Xu et al. [127] obtained SCS-based transcriptome profiles of single cells from primary tumors, negative lymph nodes (NL), and positive lymph nodes (PL). They also performed a single-cell assay for transposase-accessible chromatin (ATAC) sequencing (scATAC-seq) of the P- and NL samples. The result showed a novel cell subpopulation with an abnormally high expression level of CXCL14 in the PL of breast cancer patients. Integrative analyses of scRNA-seq and scATAC-seq revealed CXCL14 as a key regulator of lymph node metastasis in breast cancer. Lawson et al. [128] analyzed the gene expression of metastatic breast cancer at different stages by SCS and found that gene expression levels of metastatic tumor cells in early lesions were significantly different than that of the primary lesions. Moreover, they also found a small number of stem cell-like clonal subpopulations in primary tumors and detected the expression of various stem cell genes in metastatic tumor cells with early pathological changes, confirming that these cells can differentiate into coelomic metastatic cells, thus clarifying the role of stem cell-like clonal subpopulations in breast cancer metastasis. In summary, it is necessary to find the corresponding targets for drugs focusing on cell subsets or genes with metastatic tendency to enable targeted treatment and improve the prognosis of early and middle stage cancer patients.

**Table 3:** Potential application of single-cell sequencing in human cancers

Tumor type	Sample type	Number	Detection method	Clinical significance	Ref
Colorectal cancer	Tissues	1 patient	scRNA-seq	The study found a total of 12 clusters corresponding to 6 cell types were identified from patient sample of CRC liver metastasis	[122]
Breast cancer	Tissues	–	scRNA-seq	The study elucidated role of stem cell-like clone subset in breast cancer metastasis	[128]
	Cells	55 patients	scRNA-seq ATAC-seq	This study provided a new therapeutic target for breast cancer lymph node metastasis	[127]
	Tissues	–	scRNA-seq	The study revealed the main pathway of tumor metastasis upregulation is mitochondrial oxidative phosphorylation	[80]
	Blood	5 patients	scRNA-seq	The study may provide a key therapeutic target for breast cancer metastasis	[131]
	Blood	–	scRNA-seq	The study identified a rare but highly metastatic subpopulation of CTCs	[9]
Adenocarcinoma	Cells	44 patients	scRNA-seq	The study showed that ts2-specific related genes are associated with lung adenocarcinoma progression and metastasis	[41]
Pancreatic cancer	Blood	168 single CTCs	scRNA-seq	The study found the SPARC gene is highly expressed in pancreatic CTCs, which may provide a novel target for the therapy of pancreatic cancer	[132]
Prostate cancer	Tissues	10 patients	scRNA-seq	The study showed that mutations in androgen receptor signaling reveals unprecedented, detailed transfer mechanisms	[124]
Metastatic melanoma	Tissues	19 patients	scRNA-seq	The study showed the mechanism of T lymphocyte activation and cloning in this tumor tissues	[129]
Clear cell renal cell carcinoma	Tissues	121 cells	scRNA-seq	The study found and identified 44 metastasis-associated marker genes	[130]

SCS can also identify the details of metastasis mechanism, which is expected to provide therapeutic targets for tumor metastasis treatment. Gundem et al. [124] used SCS to characterize multiple metastatic tissues caused by prostate cancer in ten patients and comprehensively analyzed the characteristics of the subclonal system. It was found that AR signal transduction gene mutations are usually found in different metastases, revealing an unprecedented detail on metastatic mechanism, further elucidating the mechanism of metastasis to distant organs. It is worth mentioning that SCS can also explain the metabolic theory of metastatic cancer. Davis et al. [80] highlighted that breast cancer cells use mitochondrial metabolism during metastasis, and that those drugs targeting mitochondria can effectively prevent cancer cell metastasis. The authors also analyzed the transcriptome profiles of primary breast cancer and its micrometastases by SCS and determined that the main mechanism of upregulation of tumor metastasis is mitochondrial oxidative phosphorylation (OXPHOS). This indicates that selective inhibition of OXPHOS might be a novel targeted therapy strategy to prevent breast cancer metastasis. Using SCS to study the composition of immune cell groups in the tumor environment will help identify promising targets for cancer immunotherapy. Zhang et al. [122] used SCS to analyze samples of liver metastasis tissue and adjacent tissues from colorectal cancer patients to further study the microenvironment composition and characteristics of liver metastasis of colorectal carcinoma. Twelve clusters corresponding to six cell types were identified. The clinical significance of 93 cell cluster-specific disorder genes (CCSDGs) in tumor-infiltrating immunocytes was discussed. The Wnt signaling pathway was found to be activated and promoted granulocyte migration. SCS analysis may also help shed light on TME composition and mechanisms of CRC liver metastasis. Similarly, SCS analysis of tumor-infiltrating T lymphocytes in the metastatic melanoma microenvironment revealed T cell activation and clonal expansion in the tumor tissue [129]. Single-cell genomics offers further insights with implications for both targeted and immune therapies. Zhang et al. [130] used SCS to decipher the tumor heterogeneity of all cell subsets, including clear renal cell carcinoma (ccRCC). The authors characterized 121 cell samples. They found 44 metastasis-related marker genes and verified 14 key metastasis-related genes (MAGs), which confirmed that MAGs were related to multiple risk prognosis. In addition, patients with high MAGs nomogram scores were related to the upregulation of oxidative phosphorylation, Wnt signaling pathway, and MAPK signaling pathway in ccRCC. SCS may thus be valuable to identify potential drug targets in metastatic ccRCC.

With the continuous advancement of SCS technologies and the standardization of CTC enrichment and identification, SCS of CTCs can be used to compare the differences in the genetic make-up, transcription, and epigenome of single cells in primary and metastatic tumors and metastatic lymph nodes, and thus to determine potential treatment targets and discover transfer-related cell subsets or mutated genes. Aceto et al. [131] analyzed CTCs from mouse models with tagged breast cancer using SCS and found that rare CTC clusters exhibit increased metastatic potential compared to single CTCs. In addition, RNA sequencing of CTC clusters of human breast cancer confirmed the key role of plakoglobin in the formation of tumor cell clusters. In mouse models, plakoglobin knockdown abrogates CTC cluster formation and suppresses lung metastases, indicating that plakoglobin may be a critical therapeutic target for metastatic breast cancer. Ting et al. [132] separated CTCs from a pancreatic cancer mouse model, analyzed the whole-genome expression profile of single CTCs, and matched primary tumors by SCS. The extracellular matrix (ECM) associated gene secreted protein acidic and cysteine rich (SPARC) was found highly expressed in mouse and human pancreatic cancer CTCs, and this gene has also been proven to be closely related to pancreatic cancer metastasis. Knocking out *SPARC* can inhibit cell migration and invasion. Thus, *SPARC* is expected to become a new target for pancreatic cancer treatment.

The development of AI industry enables easier and more visually appealing solutions for SCS technology. For example, AI can be widely exploited in all aspects of the SCS workflow, such as batch correction for technical heterogeneity [133, 134], feature extraction [135, 136], data distribution transformation [137, 138], classification of cancer subtypes [139, 140], and biomarker identification [141–143]. Most notably, SCS in combination with AI is also widely used to identify and analyze CTCs, a class of cells that can be used for searching therapeutic targets for tumor metastasis [133–144]. For instance, AI-based cell identification technology “Deepcell” can be used for morphological identification of living cells to assist single-cell genomics and liquid biopsy [140]. Furthermore, MagRC, a new AI technology, is able to distinguish CTCs in whole blood cells and classify the heterogeneous CTCs [145]. Such combined use with AI enables a more comprehensive analysis of CTCs, is not influenced by interference between operators, and therefore is expected to be an essential tool to identify metastasis-related markers and therapeutic targets. Thus, combined with AI, SCS will be the pivotal tool for exploiting the information available in genomic big data and ultimately “deliver” therapy of precision.

In conclusion, SCS can be used to mine the therapeutic targets related to tumor metastasis and provide a theoretical basis for basic researchers to find potential drug therapeutic targets. With the combined application of AI, the mining of CTCs has become in-depth and more convenient.

### Monitoring of treatment response and optimization of treatment strategy

Tumor heterogeneity poses a complex challenge to cancer treatment and is a critical determinant of treatment response and metastasis. Metastatic tumors are different from primary tumors in terms of cell proliferation rate, invasion, and metastasis ability, which in turn lead to differences in therapy response and prognosis. Malignant ascites can be caused by metastasis of various cancers, including CRC. Poonpanichakul et al. [146] used SCS to explore and characterize 19,653 ascites-derived cells from four patients with CRC. Unbiased clustering of these cells revealed 14 subgroups with unique transcriptome patterns in four main cell types: epithelial and bone marrow cells, fibroblasts, and lymphocytes. Analysis of epithelial cell subsets showed that only three of the eleven subsets contracted significantly after treatment, indicating that most of the heterogeneous ascites-derived cells were resistant to treatment. Hence, a highly heterogeneous cancer subgroup at the single-cell level was determined. In other words, different cell types responded differently to chemotherapy. Overall, this study highlighted the potential benefits of SCS in real-time monitoring of the treatment response of cancer patients. Studying the phenotype of the primary tumor alone may lead to poor treatment choices. Early detection and characterization of the CTC phenotype can help optimize drug treatment strategy and monitor treatment response [147], as the identified gene expression characteristics of the CTCs are related to the treatment response and metastasis risk of lung [148], breast [149, 150], and prostate cancers [151, 152]. Miyamoto et al. [152] analyzed 77 CTCs in the peripheral blood of prostate cancer patients using SCS and found heterogeneity in gene expression in CTCs. Based on this finding, therapeutic response to androgen receptor (AR) inhibitors in patients was retrospectively analyzed. The results showed that the cell-signaling pathway in CTCs was affected in patients who received AR inhibitor treatment. The authors attributed this finding to the treatment response of patients, and SCS was indeed shown to reflect the treatment responses of tumors. Su et al. [148] used SCS to track and analyze copy number alterations (CNA) of CTCs in small-cell lung cancer (SCLC) at different time points during treatment and found that the patient's survival could be predicted based on the initial CNA score prior to treatment. Accordingly, lower CNA scores indicate longer survival times and better treatment responses. Thus, monitoring of the CNA scores of CTCs at different time points during chemotherapy can be used to evaluate treatment responses. Shih et al. [153] studied primary and metastatic tumor tissue samples from high-grade serous ovarian cancer patients through high-throughput SCS analysis. According to their results, *CD24*, *EPCAM*, and *KRT18* genes were significantly expressed in epithelial cells of primary tumors, whereas the corresponding metastatic lesions showed high expressions of *CD44* in T and B cell clusters. Elevated *CD44* expression was previously shown to be an independent prognostic indicator of shorter overall survival (OS) in serous ovarian cancer patients [154]. Schulz et al. [155] analyzed changes in microglia and blood-derived monocytes in the microenvironment of brain metastasis using SCS and revealed cellular and molecular changes in the medullary compartment at different brain metastasis stages and response to radiotherapy, which indicated that SCS can also be used to monitor the therapeutic response in brain metastasis. Another potential application of SCS may be the investigation of tumor-derived exosomes (TEXs). Exosomes, a class of small extracellular vesicles, are associated with biological phenomena, such as tumor metastasis and treatment response [156]. SCS can be used for RNA sequencing in TEXs, and thus for the longitudinal monitoring of the RNA expression profile in circulating exosomes and studying the changes in immune pathway genes during the course of immunotherapy and the differential expression patterns between responders and non-responders [157]. The potential of the SCS technique to characterize these complicated microvesicles is promising. Fathi et al. [158] confirmed that pathways related to extracellular vesicle (EV) secretion were enriched in the non-metastatic cells (compared with metastatic cells) using SCS. Analysis of the results from in vitro experiments and animal studies with results obtained using these cell lines suggested that tumors enriched in CD81 + CD63 + EV signatures have a better prognosis compared with tumors with fewer CD81 + CD63 + EVs signatures in non-metastatic breast tumors. It can be seen that studying TEXs can help to monitor the treatment response and infer the prognosis of tumor patients.

SCS can also be used to identify novel markers that can predict treatment response. Wang et al. [159] investigated tumor heterogeneity in dense and loose pancreatic ductal adenocarcinoma (PDAC) using SCS and found that PDAC patients with abundant meCAFs had a higher metastasis risk and a poor prognosis, yet showed better response to immunotherapy. This indicates that the new CAF subtype can be used as a biomarker for treatment response prediction. Gastric adenocarcinoma (GAC) tumor cells metastasize to peritoneal carcinoma (PC), but the basic mechanisms of peritoneal carcinomatosis are currently unclear. Wang et al. [160] sequenced whole exons and transcriptomes of 44 PC patients, and identified two main molecular subtypes of PC, namely “epithelioid” and “mesenchymal-like,” which show different responses to chemotherapy, and can thus be used to predict the therapeutic response. Fairfax et al. [161] characterized the gene expression of CD8 + T cells in a group of metastatic melanoma patients treated with checkpoint blockers using SCS. Their results showed that CD8 + T clones in peripheral blood could be used to predict long-term response to checkpoint blocking. Drug sensitivity experiments based on predictive metastasis-related factors were also conducted to predict treatment response. Clear cell renal cell carcinoma (ccRCC) is the most common form of renal cell carcinoma. Kim et al. [162] applied SCS to examine the intratumoral heterogeneity of a pair of primary renal cell carcinomas and their pulmonary metastases. They found that *EGFR* and *SRC* could be considered as target genes based on their high expression levels for combined targeting treatment in metastatic renal cell carcinoma. In addition, drug sensitivity of single tumor cells was also predicted, and four metastatic renal cell carcinoma (mRCC) subsets with different drug sensitivities and signaling pathway activation profiles were identified. Finally, a combinatorial strategy regimen was predicted, which targeted two mutually exclusive pathways. In this strategy, metastatic cancer cells were derived based on the activation of multiple drug target pathways. Thus, a combinatorial therapeutic strategy was shown to be superior to monotherapy in metastatic renal cell carcinoma.

These studies show the potential of SCS at monitoring and predicting the treatment responses of metastatic tumors, as well as the screening of novel molecular markers to further optimize clinical drug treatment options.

## Challenges

Although great progress has been made in the field of SCS, this technique remains challenging and is far from being used routinely [163, 164] due to the following factors: (1) single-cell collection is tricky. A small amount of sample material is used, but analysis still requires a sufficient number of cells to ensure that all cell types are labeled; (2) sample separation method and storage are not yet fully and comprehensively established. The separation technology may cause cellular injury; thus, careful operation and practical experience are required; (3) there are differences in the quality and efficiency of amplification products. When different sequencing platforms detect the same sample, owing to the fact that the PCR amplification efficiency of each platform is different, the results will be different. In addition, as the amount of DNA or mRNA contained in each cell is very small, it is necessary to perform a whole-genome or whole-transcriptome amplification step first. It is mainly manifested in two aspects: (i) it is difficult to achieve true genome-wide amplification. As a result, some regions in the genome are amplified and some are not, and the regions that have not been amplified cannot be sequenced; (ii) the gene expression levels in the two samples are the same, but the amplification efficiency is inconsistent. After N cycles of amplification, the expression profile of the two samples after amplification will be very different. When analyzing the differential genes, if 1.5 times is selected as the standard for differential genes, then there may be false differences between genes; (4) the technology is expensive. The use of SCS has been limited in part because of its high cost and long operating time. Most of the instruments and reagents required are expensive; (5) the analysis of SCS data is too difficult. As the scale of the experiment increases, the burden of data analysis also increases. Moreover, when there is too much data, the computer runs slowly and it is inconvenient to download and save. The potential of SCS technology for large-scale application in clinical diagnosis, treatment guidance, and treatment monitoring remains to be further tested in the face of these limitations. All in all, we expect that these bottlenecks will be overcome in the near future with technological advancements.

## Conclusions and prospects

In 2011, the journal Nature Methods listed SCS as one of the emerging technologies worth looking forward to. In 2013, Science listed SCS as the most noteworthy technology of the year. In 2018, SCS ranked first among the top ten scientific breakthroughs in Science once again. Several research institutions cooperated to characterize a human tumor map network (HTAN) based on SCS data in 2020 [165]. Furthermore, the literature shows that SCS has unlimited potential for application in various research fields such as basic scientific research and clinical medicine in the future and will affect the direction of future scientific development. This review shows that SCS technique has excellent application potential with respect to research on tumor metastasis. It can be used to draw comprehensive maps of single tumor cells and accurately compare the heterogeneity of different tumor cells, such as those from primary and metastatic tumors, for predicting and monitoring tumor metastasis, clarifying metastasis mechanisms, identifying therapeutic targets, monitoring and predicting the therapeutic response, and optimizing treatment strategies. The most promising application of SCS is analyzing tumor metastasis through the identification of CTCs. Use of SCS in combination with AI to identify CTCs and mechanisms underlying tumor metastasis is the “icing on the cake.” In conclusion, SCS has great prospects with respect to conquering tumor metastasis, and is expected to provide new therapeutic targets for tumor metastasis [166].

## Data Availability

Data sharing is not applicable to this article as no datasets were generated or analyzed during the current study.

## Abbreviations

SCS: Single-cell sequencing
scDNA-seq: Single-cell DNA sequencing
scRNA-seq: Single-cell RNA sequencing
scIR-seq: Single-cell immune repertoire sequencing. COVID-19: coronavirus disease 2019
TME: Tumor microenvironment
TIME: Tumor immune microenvironment
EMT: Epithelial–mesenchymal transition
MET: Mesenchymal–epithelial transition
CAFs: Cancer-related fibroblasts
CTCs: Circulating tumor cells
CSCs: Cancer stem cells
TAMs: Tumor-related macrophages
ECM: Extracellular matrix
BBB: Blood–brain barrier
BTB: Blood–tumor barrier
CAN: Copy number alterations
MAPK: Mitogen-activated protein kinase
SPARC: Secreted protein acidic and cysteine rich
LCP1: Lymphocyte cytoplasmic protein 1
PC: Peritoneal carcinoma
GAC: Gastric adenocarcinoma
P-Gp: P-glycoprotein
MDR: Multidrug resistance
AI: Artificial intelligence
mRCC: Metastatic renal cell carcinoma
ER: Estrogen receptor
PR: Progesterone receptor
HER2: Human epidermal growth factor
AR: Androgen receptor

## References

[1] Chaffer CL, Weinberg RA (2011). A perspective on cancer cell metastasis. Science.

[2] Zhou H, He X, He Y, Ou C, Cao P (2021). Exosomal circRNAs: emerging players in tumor metastasis. Front Cell Dev Biol.

[3] Siegel RL, Miller KD, Jemal A (2020). Cancer statistics, 2020. CA Cancer J Clin.

[4] Harper KL, Sosa MS, Entenberg D, Hosseini H, Cheung JF, Nobre R (2016). Mechanism of early dissemination and metastasis in Her2(+) mammary cancer. Nature.

[5] Hosseini H, Obradovic MMS, Hoffmann M, Harper KL, Sosa MS, Werner-Klein M (2016). Early dissemination seeds metastasis in breast cancer. Nature.

[6] Hu Z, Curtis C (2020). Looking backward in time to define the chronology of metastasis. Nat Commun.

[7] Tang X, Huang Y, Lei J, Luo H, Zhu X (2019). The single-cell sequencing: new developments and medical applications. Cell Biosci.

[8] Perone Y, Farrugia AJ, Rodríguez-Meira A, Győrffy B, Ion C, Uggetti A (2019). SREBP1 drives Keratin-80-dependent cytoskeletal changes and invasive behavior in endocrine-resistant ERα breast cancer. Nat Commun.

[9] Chen YC, Sahoo S, Brien R, Jung S, Humphries B, Lee W (2019). Single-cell RNA-sequencing of migratory breast cancer cells: discovering genes associated with cancer metastasis. Analyst.

[10] Bartoschek M, Oskolkov N, Bocci M, Lovrot J, Larsson C, Sommarin M (2018). Spatially and functionally distinct subclasses of breast cancer-associated fibroblasts revealed by single cell RNA sequencing. Nat Commun.

[11] Andrews TS, Kiselev VY, McCarthy D, Hemberg M (2021). Tutorial: guidelines for the computational analysis of single-cell RNA sequencing data. Nat Protoc.

[12] Zhang Y, Wang D, Peng M, Tang L, Ouyang J, Xiong F (2021). Single-cell RNA sequencing in cancer research. J Exp Clin Cancer Res.

[13] Li L, Xiong F, Wang Y, Zhang S, Gong Z, Li X (2021). What are the applications of single-cell RNA sequencing in cancer research: a systematic review. J Exp Clin Cancer Res.

[14] Hwang B, Lee JH, Bang D (2018). Single-cell RNA sequencing technologies and bioinformatics pipelines. Exp Mol Med.

[15] Tang F, Barbacioru C, Wang Y, Nordman E, Lee C, Xu N (2009). mRNA-Seq whole-transcriptome analysis of a single cell. Nat Methods.

[16] Islam S, Kjallquist U, Moliner A, Zajac P, Fan JB, Lonnerberg P (2011). Characterization of the single-cell transcriptional landscape by highly multiplex RNA-seq. Genome Res.

[17] Ramsköld D, Luo S, Wang YC (2012). Full-length mRNA-Seq from single-cell levels of RNA and individual circulating tumor cells[J]. Nat Biotechnol.

[18] Picelli S, Bjorklund AK, Faridani OR, Sagasser S, Winberg G, Sandberg R (2013). Smart-seq2 for sensitive full-length transcriptome profiling in single cells. Nat Methods.

[19] Azizi E, Carr AJ, Plitas G, Cornish AE, Konopacki C, Prabhakaran S (2018). Single-Cell Map of Diverse Immune Phenotypes in the Breast Tumor Microenvironment. Cell.

[20] Yilmaz S, Singh AK (2012). Single cell genome sequencing. Curr Opin Biotechnol.

[21] Zhuo W, Xiaohan S, Qihui S (2021). Advances in single-cell whole genome sequencing technology and its application in biomedicine. Yi Chuan.

[22] Bai X, Li Y, Zeng X, Zhao Q, Zhang Z (2021). Single-cell sequencing technology in tumor research. Clin Chim Acta.

[23] Yaari G, Kleinstein SH (2015). Practical guidelines for B-cell receptor repertoire sequencing analysis. Genome Med.

[24] Tian Y, Carpp LN, Miller HER, Zager M, Newell EW, Gottardo R (2022). Single-cell immunology of SARS-CoV-2 infection. Nat Biotechnol.

[25] Hashimshony T, Senderovich N, Avital G, Klochendler A, de Leeuw Y, Anavy L (2016). CEL-Seq2: sensitive highly-multiplexed single-cell RNA-Seq. Genome Biol.

[26] Zheng GX, Terry JM, Belgrader P, Ryvkin P, Bent ZW, Wilson R (2017). Massively parallel digital transcriptional profiling of single cells. Nat Commun.

[27] Macosko EZ, Basu A, Satija R, Nemesh J, Shekhar K, Goldman M (2015). Highly Parallel Genome-wide Expression Profiling of Individual Cells Using Nanoliter Droplets. Cell.

[28] Gierahn TM, Wadsworth MH, Hughes TK, Bryson BD, Butler A, Satija R (2017). Seq-Well: portable, low-cost RNA sequencing of single cells at high throughput. Nat Methods.

[29] Klein AM, Mazutis L, Akartuna I, Tallapragada N, Veres A, Li V (2015). Droplet barcoding for single-cell transcriptomics applied to embryonic stem cells. Cell.

[30] Yanai I, Hashimshony T (2019). CEL-Seq2-single-cell RNA sequencing by multiplexed linear amplification. Methods Mol Biol.

[31] Ziegenhain C, Vieth B, Parekh S, Reinius B, Guillaumet-Adkins A, Smets M (2017). Comparative analysis of single-cell RNA sequencing methods. Mol Cell.

[32] Zhang X, Li T, Liu F, Chen Y, Yao J, Li Z (2019). Comparative analysis of droplet-based ultra-high-throughput single-cell RNA-Seq systems. Mol Cell.

[33] Aicher TP, Carroll S, Raddi G, Gierahn T, Wadsworth MH, Hughes TK (2019). Seq-Well: a sample-efficient, portable picowell platform for massively parallel single-cell RNA sequencing. Methods Mol Biol.

[34] Zilionis R, Nainys J, Veres A, Savova V, Zemmour D, Klein AM (2017). Single-cell barcoding and sequencing using droplet microfluidics. Nat Protoc.

[35] Choi JR, Yong KW, Choi JY, Cowie AC (2020). Single-cell RNA sequencing and its combination with protein and DNA analyses. Cells.

[36] Fu J, Akat KM, Sun Z, Zhang W, Schlondorff D, Liu Z (2019). Single-cell RNA profiling of glomerular cells shows dynamic changes in experimental diabetic kidney disease. J Am Soc Nephrol.

[37] Zhang F, Wei K, Slowikowski K, Fonseka CY, Rao DA, Kelly S (2019). Defining inflammatory cell states in rheumatoid arthritis joint synovial tissues by integrating single-cell transcriptomics and mass cytometry. Nat Immunol.

[38] Farbehi N, Patrick R, Dorison A, Xaymardan M, Janbandhu V, Wystub-Lis K (2019). Single-cell expression profiling reveals dynamic flux of cardiac stromal, vascular and immune cells in health and injury. Elife.

[39] Farrell JA, Wang Y, Riesenfeld SJ, Shekhar K, Regev A, Schier AF (2018). Single-cell reconstruction of developmental trajectories during zebrafish embryogenesis. Science..

[40] Wang J, Xu Y, Chen Z, Liang J, Lin Z, Liang H (2020). Liver immune profiling reveals pathogenesis and therapeutics for biliary atresia. Cell.

[41] Su T, Yang Y, Lai S, Jeong J, Jung Y, McConnell M (2021). Single-cell transcriptomics reveals zone-specific alterations of liver sinusoidal endothelial cells in cirrhosis. Cell Mol Gastroenterol Hepatol.

[42] Khateb M, Azriel A, Levi BZ (2019). The third intron of IRF8 is a cell-type-specific chromatin priming element during mouse embryonal stem cell differentiation. J Mol Biol.

[43] Wilk AJ, Rustagi A, Zhao NQ, Roque J, Martinez-Colon GJ, McKechnie JL (2020). A single-cell atlas of the peripheral immune response in patients with severe COVID-19. Nat Med.

[44] Kim N, Kim HK, Lee K, Hong Y, Cho JH, Choi JW, Lee JI, Suh YL, Ku BM, Eum HH (2020). Single-cell RNA sequencing demonstrates the molecular and cellular reprogramming of metastatic lung adenocarcinoma. Nat Commun.

[45] Zheng L, Qin S, Si W, Wang A, Xing B, Gao R, Ren X, Wang L, Wu X, Zhang J (2012). Pan-cancer single-cell landscape of tumor-infiltrating T cells. Science.

[46] Fares J, Fares MY, Khachfe HH, Salhab HA, Fares Y (2020). Molecular principles of metastasis: a hallmark of cancer revisited. Signal Transduct Target Ther.

[47] Zeeshan R, Mutahir Z (2017). Cancer metastasis - tricks of the trade. Bosn J Basic Med Sci.

[48] Hunter KW, Amin R, Deasy S, Ha NH, Wakefield L (2018). Genetic insights into the morass of metastatic heterogeneity. Nat Rev Cancer.

[49] Wu XX, Yue GG, Dong JR, Lam CW, Wong CK, Qiu MH (2020). Actein Inhibits tumor growth and metastasis in HER2-positive breast tumor bearing mice via suppressing AKT/MTOR and RAS/RAF/MAPK signaling pathways. Front Oncol.

[50] Jin W (2020). Role of JAK/STAT3 signaling in the regulation of metastasis, the transition of cancer stem cells, and chemoresistance of cancer by epithelial-mesenchymal transition. Cells.

[51] Ge X, Liu W, Zhao W, Feng S, Duan A, Ji C (2020). Exosomal transfer of LCP1 promotes osteosarcoma cell tumorigenesis and metastasis by activating the JAK2/STAT3 signaling pathway. Mol Ther Nucleic Acids.

[52] Zhan T, Rindtorff N, Boutros M (2017). Wnt signaling in cancer. Oncogene.

[53] Zhu HH, Zhu XY, Zhou MH, Cheng GY, Lou WH (2014). Effect of WNT5A on epithelial-mesenchymal transition and its correlation with tumor invasion and metastasis in nasopharyngeal carcinoma. Asian Pac J Trop Med.

[54] Yu M, Ting DT, Stott SL, Wittner BS, Ozsolak F, Paul S (2012). RNA sequencing of pancreatic circulating tumour cells implicates WNT signalling in metastasis. Nature.

[55] Lin X, Xiaoqin H, Jiayu C, Li F, Yue L, Ximing X (2019). Long non-coding RNA miR143HG predicts good prognosis and inhibits tumor multiplication and metastasis by suppressing mitogen-activated protein kinase and Wnt signaling pathways in hepatocellular carcinoma. Hepatol Res.

[56] Meacham CE, Morrison SJ (2013). Tumour heterogeneity and cancer cell plasticity. Nature.

[57] Zhang F, Zhang H, Wang Z, Yu M, Tian R, Ji W (2014). P-glycoprotein associates with Anxa2 and promotes invasion in multidrug resistant breast cancer cells. Biochem Pharmacol.

[58] Tomono T, Yano K, Ogihara T (2017). Snail-induced epithelial-to-mesenchymal transition enhances P-gp-mediated multidrug resistance in HCC827 cells. J Pharm Sci.

[59] Zhang HC, Zhang F, Wu B, Han JH, Ji W, Zhou Y (2012). Identification of the Interaction between P-Glycoprotein and Anxa2 inMultidrug-resistant human breast cancer cells. Cancer Biol Med.

[60] Ribelles N, Santonja A, Pajares B, Llacer C, Alba E (2014). The seed and soil hypothesis revisited: current state of knowledge of inherited genes on prognosis in breast cancer. Cancer Treat Rev.

[61] Hoshino A, Costa-Silva B, Shen TL, Rodrigues G, Hashimoto A, Tesic Mark M (2015). Tumour exosome integrins determine organotropic metastasis. Nature.

[62] Ganesh K, Massague J (2021). Targeting metastatic cancer. Nat Med.

[63] Zhang HG, Grizzle WE (2014). Exosomes: a novel pathway of local and distant intercellular communication that facilitates the growth and metastasis of neoplastic lesions. Am J Pathol.

[64] Pastushenko I, Blanpain C (2019). EMT transition states during tumor progression and metastasis. Trends Cell Biol.

[65] Calon A, Lonardo E, Berenguer-Llergo A, Espinet E, Hernando-Momblona X, Iglesias M (2015). Stromal gene expression defines poor-prognosis subtypes in colorectal cancer. Nat Genet.

[66] Lamouille S, Xu J, Derynck R (2014). Molecular mechanisms of epithelial-mesenchymal transition. Nat Rev Mol Cell Biol.

[67] Lin Y, Xu J, Lan H (2019). Tumor-associated macrophages in tumor metastasis: biological roles and clinical therapeutic applications. J Hematol Oncol.

[68] Fu LQ, Du WL, Cai MH, Yao JY, Zhao YY, Mou XZ (2020). The roles of tumor-associated macrophages in tumor angiogenesis and metastasis. Cell Immunol.

[69] Wang D, Wang X, Si M, Yang J, Sun S, Wu H (2020). Exosome-encapsulated miRNAs contribute to CXCL12/CXCR4-induced liver metastasis of colorectal cancer by enhancing M2 polarization of macrophages. Cancer Lett.

[70] Li K, Kang H, Wang Y, Hai T, Rong G, Sun H (2016). Letrozole-induced functional changes in carcinoma-associated fibroblasts and their influence on breast cancer cell biology. Med Oncol.

[71] Jayanthi P, Varun BR, Selvaraj J (2020). Epithelial-mesenchymal transition in oral squamous cell carcinoma: An insight into molecular mechanisms and clinical implications. J Oral Maxillofac Pathol.

[72] Foroni C, Broggini M, Generali D, Damia G (2012). Epithelial-mesenchymal transition and breast cancer: role, molecular mechanisms and clinical impact. Cancer Treat Rev.

[73] Bakir B, Chiarella AM, Pitarresi JR, Rustgi AK (2020). EMT, MET, plasticity, and tumor metastasis. Trends Cell Biol.

[74] Wu S, Zhang H, Fouladdel S, Li H, Keller E, Wicha MS (2020). Cellular, transcriptomic and isoform heterogeneity of breast cancer cell line revealed by full-length single-cell RNA sequencing. Comput Struct Biotechnol J.

[75] Gerlinger M, Rowan AJ, Horswell S, Larkin J, Endesfelder D, Gronroos E, Martinez P, Matthews N, Stewart A, Tarpey P, Varela I (2012). Intratumor heterogeneity and branched evolution revealed by multiregion sequencing. N Engl j Med.

[76] Leung ML, Davis A, Gao R, Casasent A, Wang Y, Sei E (2017). Single-cell DNA sequencing reveals a late-dissemination model in metastatic colorectal cancer. Genome Res.

[77] Lin W, Noel P, Borazanci EH, Lee J, Amini A, Han IW (2020). Single-cell transcriptome analysis of tumor and stromal compartments of pancreatic ductal adenocarcinoma primary tumors and metastatic lesions. Genome Med.

[78] Liu Y, Ye G, Huang L, Zhang C, Sheng Y, Wu B (2020). Single-cell transcriptome analysis demonstrates inter-patient and intra-tumor heterogeneity in primary and metastatic lung adenocarcinoma. Aging..

[79] Okamoto T, duVerle D, Yaginuma K, Natsume Y, Yamanaka H, Kusama D (2021). Comparative analysis of patient-matched PDOs revealed a reduction in OLFM4-associated clusters in metastatic lesions in colorectal cancer. Stem Cell Reports.

[80] Davis RT, Blake K, Ma D, Gabra MBI, Hernandez GA, Phung AT (2020). Transcriptional diversity and bioenergetic shift in human breast cancer metastasis revealed by single-cell RNA sequencing. Nat Cell Biol.

[81] Chaika NV, Yu F, Purohit V, Mehla K, Lazenby AJ, DiMaio D (2012). Differential expression of metabolic genes in tumor and stromal components of primary and metastatic loci in pancreatic adenocarcinoma. PLoS ONE.

[82] Zhou Y, Yang D, Yang Q, Lv X, Huang W, Zhou Z (2020). Single-cell RNA landscape of intratumoral heterogeneity and immunosuppressive microenvironment in advanced osteosarcoma. Nat Commun.

[83] Ni X, Zhuo M, Su Z, Duan J, Gao Y, Wang Z (2013). Reproducible copy numbervariation patterns among single circulating tumor cells of lung cancer patients. Proc Natl Acad Sci U S A.

[84] McGranahan N, Swanton C (2017). Clonal heterogeneity and tumor evolution: past, present, and the future. Cell.

[85] Suda K, Kim J, Murakami I, Rozeboom L, Shimoji M, Shimizu S (2018). Innate genetic evolution of lung cancers and spatial heterogeneity: analysis of treatment-naive lesions. J Thorac Oncol.

[86] Ma KY, Schonnesen AA, Brock A, Van Den Berg C, Eckhardt SG, Liu Z (2019). Single-cell RNA sequencing of lung adenocarcinoma reveals heterogeneity of immune response-related genes. JCI Insight.

[87] Zhang AW, McPherson A, Milne K, Kroeger DR, Hamilton PT, Miranda A (2018). Interfaces of Malignant and Immunologic Clonal Dynamics in Ovarian Cancer. Cell.

[88] Jimenez-Sanchez A, Memon D, Pourpe S, Veeraraghavan H, Li Y, Vargas HA (2017). Heterogeneous tumor-immune microenvironments among differentially growing metastases in an ovarian cancer patient. Cell.

[89] Pan XW, Zhang H, Xu D, Chen JX, Chen WJ, Gan SS (2020). Identification of a novel cancer stem cell subpopulation that promotes progression of human fatal renal cell carcinoma by single-cell RNA-seq analysis. Int J Biol Sci.

[90] Puram SV, Tirosh I, Parikh AS, Patel AP, Yizhak K, Gillespie S (2017). Single-cell transcriptomic analysis of primary and metastatic tumor ecosystems in head and neck cancer. Cell.

[91] Karaayvaz M, Cristea S, Gillespie SM, Patel AP, Mylvaganam R, Luo CC (2018). Unravelling subclonal heterogeneity and aggressive disease states in TNBC through single-cell RNA-seq. Nat Commun.

[92] Yates LR, Knappskog S, Wedge D, Farmery JHR, Gonzalez S, Martincorena I (2017). Genomic Evolution of Breast Cancer Metastasis and Relapse. Cancer Cell.

[93] Navin N, Kendall J, Troge J, Andrews P, Rodgers L, McIndoo J (2011). Tumour evolution inferred by single-cell sequencing. Nature.

[94] Saunders NA, Simpson F, Thompson EW, Hill MM, Endo-Munoz L, Leggatt G (2012). Role of intratumoural heterogeneity in cancer drug resistance: molecular and clinical perspectives. EMBO Mol Med.

[95] Jordan NV, Bardia A, Wittner BS, Benes C, Ligorio M, Zheng Y (2016). HER2 expression identifies dynamic functional states within circulating breast cancer cells. Nature.

[96] Poupon R, Chazouilleres O, Balkau B, Poupon RE (1999). Clinical and biochemical expression of the histopathological lesions of primary biliary cirrhosis. UDCA-PBC Group J Hepatol.

[97] Slotman GJ, Mohit T, Raina S, Swaminathan AP, Ohanian M, Rush BF (1984). The incidence of metastases after multimodal therapy for cancer of the head and neck. Cancer.

[98] Hjortland GO, Meza-Zepeda LA, Beiske K, Ree AH, Tveito S, Hoifodt H (2011). Genome wide single cell analysis of chemotherapy resistant metastatic cells in a case of gastroesophageal adenocarcinoma. BMC Cancer.

[99] Eyler CE, Rich JN (2008). Survival of the fittest: cancer stem cells in therapeutic resistance and angiogenesis. J Clin Oncol.

[100] Nguyen A, Yoshida M, Goodarzi H, Tavazoie SF (2016). Highly variable cancer subpopulations that exhibit enhanced transcriptome variability and metastatic fitness. Nat Commun.

[101] Miyamoto DT, Zheng Y, Wittner BS, Lee RJ, Zhu H, Broderick KT (2015). RNA-Seq of single prostate CTCs implicates noncanonical Wnt signaling in antiandrogen resistance. Science.

[102] Gujral TS, Chan M, Peshkin L, Sorger PK, Kirschner MW, MacBeath G (2014). A noncanonical Frizzled2 pathway regulates epithelial-mesenchymal transition and metastasis. Cell.

[103] Iseri OD, Kars MD, Arpaci F, Atalay C, Pak I, Gunduz U (2011). Drug resistant MCF-7 cells exhibit epithelial-mesenchymal transition gene expression pattern. Biomed Pharmacother.

[104] Lee MC, Lopez-Diaz FJ, Khan SY, Tariq MA, Dayn Y, Vaske CJ (2014). Single-cell analyses of transcriptional heterogeneity during drug tolerance transition in cancer cells by RNA sequencing. Proc Natl Acad Sci USA.

[105] Prieto-Vila M, Usuba W, Takahashi RU, Shimomura I, Sasaki H, Ochiya T (2019). Single-cell analysis reveals a preexisting drug-resistant subpopulation in the luminal breast cancer subtype. Cancer Res.

[106] Nath B, Bidkar AP, Kumar V, Dalal A, Jolly MK, Ghosh SS (2019). Deciphering hydrodynamic and drug-resistant behaviors of metastatic EMT breast cancer cells moving in a constricted microcapillary. J Clin Med.

[107] Ozawa PMM, Alkhilaiwi F, Cavalli IJ, Malheiros D, de Souza Fonseca Ribeiro EM, Cavalli LR (2018). Extracellular vesicles from triple-negative breast cancer cells promote proliferation and drug resistance in non-tumorigenic breast cells. Breast Cancer Res Treat.

[108] Schmidt F, Efferth T (2016). Tumor heterogeneity, single-cell sequencing, and drug resistance. Pharmaceuticals.

[109] Grivennikov SI, Greten FR, Karin M (2010). Immunity, inflammation, and cancer. Cell.

[110] Tripathi G, Tripathi A, Johnson J, Kashyap MK (2021). Role of RNA splicing in regulation of cancer stem cell. Curr Stem Cell Res Ther..

[111] Franken A, Honisch E, Reinhardt F, Meier-Stiegen F, Yang L, Jaschinski S (2020). Detection of ESR1 mutations in single circulating tumor cells on estrogen deprivation therapy but not in primary tumors from metastatic luminal breast cancer patients. J MolDiagn.

[112] Zhang Q, Wang W, Zhou Q, Chen C, Yuan W, Liu J (2020). Roles of circRNAs in the tumour microenvironment. Mol Cancer.

[113] Sun Z, Yang S, Zhou Q, Wang G, Song J, Li Z (2018). Emerging role of exosome-derived long non-coding RNAs in tumor microenvironment. Mol Cancer.

[114] Psaila B, Lyden D (2009). The metastatic niche: adapting the foreign soil. Nat Rev Cancer.

[115] Arvanitis CD, Ferraro GB, Jain RK (2020). The blood-brain barrier and blood-tumour barrier in brain tumours and metastases. Nat Rev Cancer.

[116] Lee HO, Hong Y, Etlioglu HE, Cho YB, Pomella V, Van den Bosch B (2020). Lineage-dependent gene expression programs influence the immune landscape of colorectal cancer. Nat Genet.

[117] Robinson DR, Wu YM, Lonigro RJ, Vats P, Cobain E, Everett J (2017). Integrative clinical genomics of metastatic cancer. Nature.

[118] Li H, Courtois ET, Sengupta D, Tan Y, Chen KH, Goh JJL (2017). Referencecomponent analysis of single-cell transcriptomes elucidates cellular heterogeneity in human colorectal tumors. Nat Genet.

[119] Bao X, Shi R, Zhao T, Wang Y, Anastasov N, Rosemann M (2021). Integrated analysis of single-cell RNA-seq and bulk RNA-seq unravels tumour heterogeneity plus M2-like tumour-associated macrophage infiltration and aggressiveness in TNBC. Cancer Immunol Immunother.

[120] Winterhoff BJ, Maile M, Mitra AK, Sebe A, Bazzaro M, Geller MA (2017). Single cell sequencing reveals heterogeneity within ovarian cancer epithelium and cancer associated stromal cells. Gynecol Oncol.

[121] Lee H, Na KJ, Choi H (2021). Differences in tumor immune microenvironment in metastatic sites of breast cancer. Front Oncol.

[122] Zhang Y, Song J, Zhao Z, Yang M, Chen M, Liu C (2020). Single-cell transcriptome analysis reveals tumor immune microenvironment heterogenicity and granulocytes enrichment in colorectal cancer liver metastases. Cancer Lett.

[123] Kuipers J, Jahn K, Beerenwinkel N (2017). Advances in understanding tumour evolution through single-cell sequencing. Biochim Biophys Acta Rev Cancer.

[124] Gundem G, Van Loo P, Kremeyer B, Alexandrov LB, Tubio JMC, Papaemmanuil E (2015). The evolutionary history of lethal metastatic prostate cancer. Nature.

[125] Han K, Wang F-W, Cao C-H, Ling H, Chen J-W, Chen R-X (2020). CircLONP2 enhances colorectal carcinoma invasion and metastasis through modulating the maturation and exosomal dissemination of microRNA-17. Molecular Cancer..

[126] Maynard A, McCoach CE, Rotow JK, Harris L, Haderk F, Kerr DL (2020). Therapy-induced evolution of human lung cancer revealed by single-cell RNA sequencing. Cell.

[127] Xu K, Zhang W, Wang C, Hu L, Wang R, Wang C (2021). Integrative analyses of scRNA-seq and scATAC-seq reveal CXCL14 as a key regulator of lymph node metastasis in breast cancer. Hum Mol Genet.

[128] Lawson DA, Bhakta NR, Kessenbrock K, Prummel KD, Yu Y, Takai K (2015). Single-cell analysis reveals a stem-cell program in human metastatic breast cancer cells. Nature.

[129] Tirosh I, Izar B, Prakadan SM, Wadsworth MH, Treacy D, Trombetta JJ (2016). Dissecting the multicellular ecosystem of metastatic melanoma by single-cell RNA-seq. Science.

[130] Zhang C, He H, Hu X, Liu A, Huang D, Xu Y, Chen L, Xu D (2019). Development and validation of a metastasis-associated prognostic signature based on single-cell RNA-seq in clear cell renal cell carcinoma. Aging.

[131] Aceto N, Bardia A, Miyamoto DT, Donaldson MC, Wittner BS, Spencer JA (2014). Circulating tumor cell clusters are oligoclonal precursors of breast cancer metastasis. Cell.

[132] Ting DT, Wittner BS, Ligorio M, Vincent Jordan N, Shah AM, Miyamoto DT (2014). Single-cell RNA sequencing identifies extracellular matrix gene expression by pancreatic circulating tumor cells. Cell Rep.

[133] Shaham U, Stanton KP, Zhao J, Li H, Raddassi K, Montgomery R, Kluger Y (2017). Removal of batch effects using distributionmatching residual networks. Bioinformatics.

[134] Li X, Wang K, Lyu Y, Pan H, Zhang J, Stambolian D, Susztak K, Reilly MP, Hu G, Li M (2020). Deep learning enables accurate clustering with batch effect removal in single-cell RNA-seq analysis. Nat Commun.

[135] Elbashir MK, Ezz M, Mohammed M, Saloum SS (2019). Lightweight convolutional neural network for breast cancer classification using RNA-seq gene expression data. IEEE Access.

[136] Ding J, Condon A, Shah SP (2018). Interpretable dimensionality reduction of single cell transcriptome data with deep generative models. Nat Commun.

[137] Barbie DA, Tamayo P, Boehm JS, Kim SY, Moody SE, Dunn IF, Schinzel AC, Sandy P, Meylan E, Scholl C (2009). Systematic RNA interference reveals that oncogenic KRAS-driven cancers require TBK1. Nature.

[138] Lauria A, Peirone S, Giudice MD, Priante F, Rajan P, Caselle M, Oliviero S, Cereda M (2020). Identification of altered biological processes in heterogeneous RNA-sequencing data by discretization of expression profiles. Nucleic Acids Res.

[139] Chen R, Yang L, Goodison S, Sun Y (2020). Deep-learning approach to identifying cancer subtypes using high-dimensional genomic data. Bioinformatics.

[140] Gao F, Wang W, Tan M, Zhu L, Zhang Y, Fessler E, Vermeulen L, Wang X (2019). DeepCC: a novel deep learning-based framework for cancer molecular subtype classification. Oncogenesis.

[141] Cheng Q, Li J, Fan F, Cao H, Dai Z-Y, Wang Z-Y, Feng S-S (2020). Identification and analysis of glioblastoma biomarkers based on single cell sequencing. Front Bioeng Biotechnol.

[142] Zhang J, Guan M, Wang Q, Zhang J, Zhou T, Sun X (2020). Single-cell transcriptome-based multilayer network biomarker for predicting prognosis and therapeutic response of gliomas. Brief Bioinform.

[143] Chen G, Ning B, Shi T (2019). Single-cell RNA-seq technologies and related computational data analysis. Front Genet.

[144] Wang Y, He X, Nie H, Zhou J, Cao P, Ou C (2020). Application of artificial intelligence to the diagnosis and therapy of colorectal cancer. Am J Cancer Res.

[145] Poudineh M, Aldridge PM, Ahmed S, Green BJ, Kermanshah L, Nguyen V, Tu C, Mohamadi RM, Nam RK, Hansen A (2017). Tracking the dynamics of circulating tumour cell phenotypes using nanoparticle-mediated magnetic ranking. Nat Nanotechnol.

[146] Poonpanichakul T, Shiao MS, Jiravejchakul N, Matangkasombut P, Sirachainan E, Charoensawan V (2021). Capturing tumour heterogeneity in pre- and post-chemotherapy colorectal cancer ascites-derived cells using single-cell RNA-sequencing. Biosci Rep..

[147] Joosse SA, Gorges TM, Pantel K (2015). Biology, detection, and clinical implications of circulating tumor cells. EMBO Mol Med.

[148] Su Z, Wang Z, Ni X, Duan J, Gao Y, Zhuo M (2019). Inferring the evolution and progression of small-cell lung cancer by single-cell sequencing of circulating tumor cells. Clin Cancer Res.

[149] Kwan TT, Bardia A, Spring LM, Giobbie-Hurder A, Kalinich M, Dubash T (2018). A digital RNA signature of circulating tumor cells predicting early therapeutic response in localized and metastatic breast cancer. Cancer Discov.

[150] Powell AA, Talasaz AH, Zhang H, Coram MA, Reddy A, Deng G (2012). Single cell profiling of circulating tumor cells: transcriptional heterogeneity and diversity from breast cancer cell lines. PLoS ONE.

[151] Lohr JG, Adalsteinsson VA, Cibulskis K, Choudhury AD, Rosenberg M, Cruz-Gordillo P (2014). Whole-exome sequencing of circulating tumor cells provides a window into metastatic prostate cancer. NatBiotechnol.

[152] Miyamoto DT, Lee RJ, Kalinich M, LiCausi JA, Zheng Y, Chen T (2018). An RNA-based digital circulating tumor cell signature is predictive of drug response and early dissemination in prostate cancer. Cancer Discov.

[153] Shih A, Menzin A, Whyte J (2018). Single-cell RNA-seq analysis of primary tumor and corresponding metastatic lesion in high-grade serous ovarian cancer. Clin Cancer Res..

[154] Ween MP, Oehler MK, Ricciardelli C (2011). Role of versican, hyaluronan and CD44 in ovarian cancer metastasis. Int J Mol Sci.

[155] Schulz M, Michels B, Niesel K, Stein S, Farin H, Rodel F (2020). Cellular and molecular changes of brain metastases-associated myeloid cells during disease progression and therapeutic response. Science.

[156] He X, Kuang G, Wu Y, Ou C (2021). Emerging roles of exosomal miRNAs in diabetes mellitus. Clin Transl Med.

[157] Wu J, Zeng D, Zhi S, Ye Z, Qiu W, Huang N, Sun L, Wang C, Wu Z, Bin J, Liao Y, Shi M, Liao W (2021). Single-cell analysis of a tumor-derived exosome signature correlates with prognosis and immunotherapy response. J Transl Med.

[158] Fathi M, Joseph R, Adolacion JRT, Martinez-Paniagua M, An X, Gabrusiewicz K, Mani SA, Varadarajan N (2021). Single-cell cloning of breast cancer cells secreting specific subsets of extracellular vesicles. Cancers (Basel).

[159] Wang Y, Liang Y, Xu H, Zhang X, Mao T, Cui J (2021). Single-cell analysis of pancreatic ductal adenocarcinoma identifies a novel fibroblast subtype associated with poor prognosis but better immunotherapy response. Cell Discov.

[160] Wang R, Song S, Harada K, Ghazanfari Amlashi F, Badgwell B, Pizzi MP (2020). Multiplex profiling of peritoneal metastases from gastric adenocarcinoma identified novel targets and molecular subtypes that predict treatment response. Gut.

[161] Fairfax BP, Taylor CA, Watson RA, Nassiri I, Danielli S, Fang H (2020). Peripheral CD8(+) T cell characteristics associated with durable responses to immune checkpoint blockade in patients with metastatic melanoma. Nat Med.

[162] Kim KT, Lee HW, Lee HO, Song HJ, da Jeong E, Shin S (2016). Application of single-cell RNA sequencing in optimizing a combinatorial therapeutic strategy in metastatic renal cell carcinoma. Genome Biol.

[163] Shapiro E, Biezuner T, Linnarsson S (2013). Single-cell sequencing-based technologies will revolutionize whole-organism science. Nat Rev Genet.

[164] Ren X, Kang B, Zhang Z (2018). Understanding tumor ecosystems by single-cell sequencing: promises and limitations. Genome Biol.

[165] Rozenblatt-Rosen O, Regev A, Oberdoerffer P, Nawy T, Hupalowska A, Rood JE (2020). The human tumor atlas network: charting tumor transitions across space and time at single-cell resolution. Cell.

[166] Théry C, Ostrowski M, Segura E (2009). Membrane vesicles as conveyors of immune responses. Nat Rev Immunol.

